# Highly Size-and Shape-Controlled Synthesis of Silver Nanoparticles via a Templated Tollens Reaction

**DOI:** 10.1002/smll.201101474

**Published:** 2012-01-09

**Authors:** Ruggero Dondi, Wu Su, Gerry A Griffith, Graham Clark, Glenn A Burley

**Affiliations:** 1 Department of Chemistry, University of LeicesterUniversity Road, Leicester, LE1 1RE,UK; 2 Department of Engineering, University of LeicesterUniversity Road, Leicester, LE1 1RE, UK; 3 Department of Pure and Applied Chemistry University of Strathclyde GlasgowG1 1XL, UK E-mail: glenn.burley@strath.ac.uk Webpage: www.burleylabs.co.uk

## Abstract

***A** mild, facile one-step synthetic strategy for the preparation of size-and shape-controlled silver nanoparticles (AgNPs) is presented. The high degree of size-and shape-control of these AgNPs is achieved by the use of triazole sugar ligands scaffolded by a central resorcinol ether core. Both the triazoles and the resorcinol ether core mediate the nucleation, growth, and passivation phases of the preparation of AgNP in the presence of the Tollens reagent as the silver source. Kinetic and ^1^H NMR titration data is presented describing the nature of the interactions between the Tollens reagent and these ligands*.

## 1. Introduction

The preparation of noble metal nanoparticle (NP) conjugates is of significant fundamental and applied importance.^1^ Owing to their unique physicochemical properties, noble metal NPs are now key players in applications ranging from nanoelectronics, medical diagnostics through to drug delivery scaffolds.^2^ As a consequence of their higher extinction coefficients relative to their gold cognate, silver nanoparticles [AgNPs] are of particular interest as highly sensitive diagnostic platforms using resonance enhancement strategies;^3^ yet the rational preparation of AgNPs with controllable dimensions remains a significant challenge.3a–c, 4 A variety of methods exist for their preparation with varying levels of success in achieving a balance between size and shape control, dispersity, stability in aqueous buffered solutions and facile preparation.3a,b Conventional methods of AgNP synthesis typically involve the reduction of silver salts with reductants of varying strengths, followed by the addition of stabilizing ligands to render the AgNPs stable in aqueous solutions suitable for biomedical applications.3a The majority of these methods however suffer from a multistep preparation, difficulty in controlling dispersity as well as their propensity to aggregate in high salt buffer solutions. There is therefore a clear need for the development of a mild, facile and reproducible synthetic strategy for the preparation of AgNPs where the variables of size and shape control, colloidal stability as well as the surface characteristics are inherently tuneable.

Of the various methods available for AgNP synthesis, the application of the Tollens-reagent [Ag(NH_3_)_2_]^+^ and reducing sugars has been under-utilized in the literature despite exhibiting distinct advantages over traditional methods.4c,e, 5 These include:

1. Mild preparative conditions–AgNP synthesis proceeds at room temperature and involves mild oxidation of an aldehyde functionality present in reducing sugars as a hemiacetal. Derivatisation of the non-anomeric sites on the sugar enables the incorporation of pendent functionality that would not withstand the typically harsh reducing environments required for other conventional preparative methods;3a and
2. Facile one-pot synthesis–Reducing sugars act both as a Ag^+^ reductants well as a passivating ligand on the AgNP surface, producing anti-bacterial AgNPs that are stable in water.4b

Previously described methods of Tollens-mediated AgNP synthesis have been confined to the utilization of widely available mono-and disaccharides. The AgNPs produced by these methods are typically ∼25–50 nm size with varying degrees of size and shape control,4b,d,e, 5b,c although the addition of magnesium salts and buffered conditions are known to produce small silver nanoclusters.^6^ We hypothesized that this varying degree of size control is primarily due to difficulties in regulating the nucleation and growth phases of the synthesis. Thus, if one could effectively regulate and indeed integrate these parameters within a single ligand system, then the production of AgNPs with programmable sizes, shapes and colloidal stabilities in buffered aqueous solutions could be achieved under mild conditions, and in a single synthetic process. To the best of our knowledge there have been no reports in the literature in which a fully integrated multifunctional ligand system has been reported for the preparation of AgNPs which:

- controls the nucleation and growth phases of the synthesis,
- passivates the surface of the resultant AgNPs, and finally
- presents functional groups on the surface that render AgNPs stable in aqueous buffered solutions.

**Figure 1:**
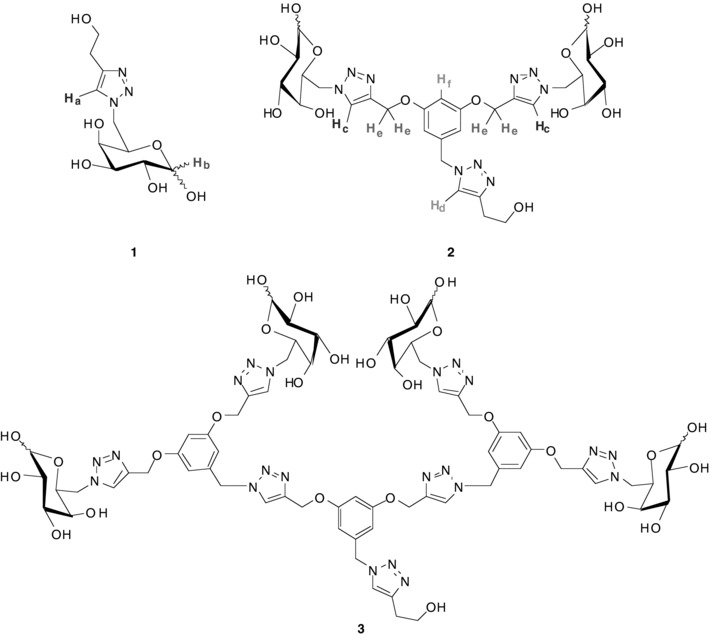
Structures of sugar triazoles (1), (2), and (3) used in the preparation of AgNPs.

In this manuscript, we present for the first time such a rationale for the preparation of size-and shape-controlled AgNPs in *a single synthetic step* via a templated reductive process using novel sugar triazole ligands (**1–3**, **Figure 1**) and the Tollens reagent as the silver source.

**Figure 2:**
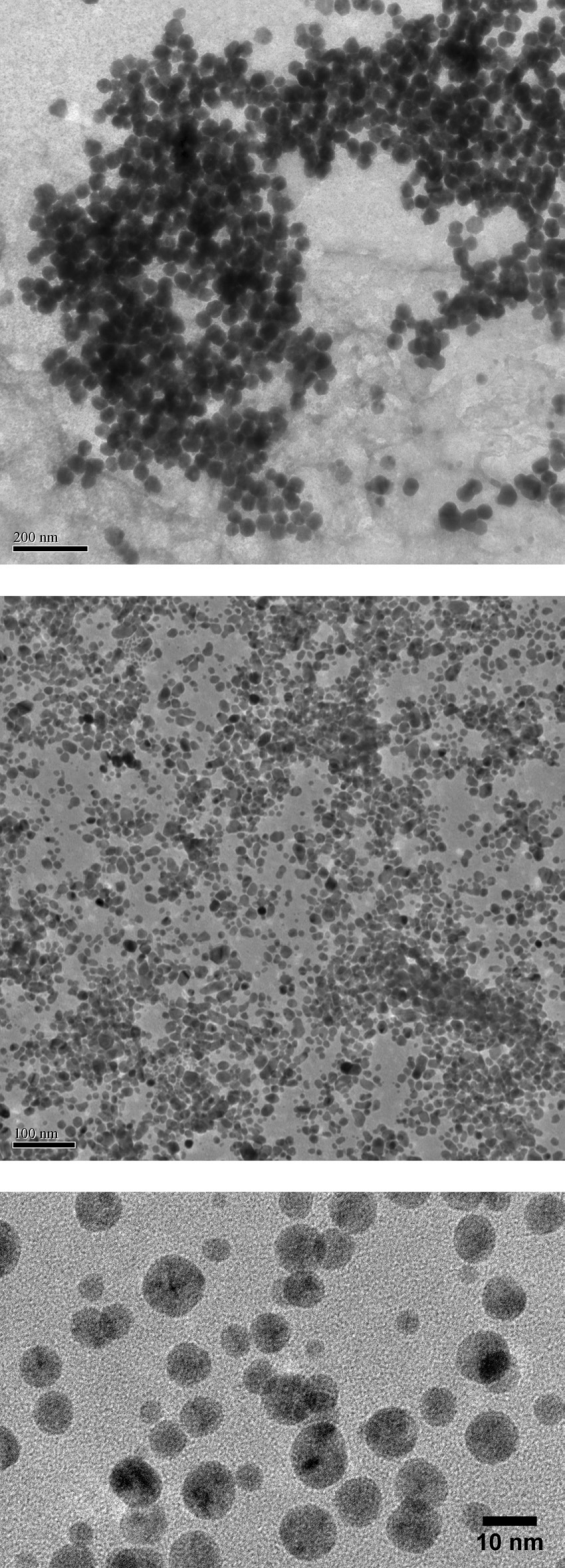
TEM images of AgNP prepared by the reduction of [Ag(NH_3_)_2_]^+^ with sugar triazole (a) (1); (b) (2), and (c) (3).

Our initial ligand design was based on a previous study which highlighted the utility of sugar triazoles for the silver staining of alkyne-modified DNA via a “click and metallization” approach.^7^ In compound (**1**), a triazole derivatized D-galactose sugar was used, whereas in (**2**) and (**3**), the D-galactose units were linked to a central resorcinol ether core via triazole linkages.^8^ We hypothesized that an increase in the number of reducing species per dendrimer would influence the size and shape control of the resulting AgNPs. In order to investigate this hypothesis, AgNP formation was screened as a function of both the concentration of the Tollens reagent [Tollens reagent] and the sugar triazoles [**1–3**]. For sugar triazole (**1**), a narrow reaction window for the formation of AgNPs [AgNP@(**1**)] was observed with a [Tollens] of 1 mM being the critical component for AgNP@(**1**) formation (Figure S1 in the Supporting Information (SI)). An optimal [Tollens reagent]: [(**1**)] ratio of 10:1 was required for the preparation of crystalline 41 ± 10 nm AgNPs prepared using (**1**) [i.e. AgNP@(**1**)] according to transmission electron microscopy (TEM) (**Figure 2**a), UV–vis spectrometry, and dynamic light scattering (DLS, Figure S2 in the SI) analysis. Tollens reagent concentrations less than 1 mM did not form appreciable amounts of AgNP@(**1**) whereas concentrations greater than 1 mM resulted in the formation of micrometer-sized aggregates and a “silver mirror” coating the reaction vessel. The shapes of AgNP@(**1**) also varied: while the majority of the particles were spherical, examples of assorted hexagonal and other polyhedra were also identified (Figure S3 in the SI), which in turn resulted in significant variation in the absorbance of the surface Plasmon ranging from 406–434 nm (Figure S2 in the SI). This size and shape dispersity of AgNPs was consistent with previous reports of AgNP formation using widely available mono-and disaccharides.4b,d,e, 5b,c

Only spherical and crystalline (face centred cubic, fcc) NPs were produced when (**2**) was used as the ligand and were significantly smaller in diameter [8 ± 5 nm] (Figure 2b and S4–S6 in teh SI) relative to the cognate AgNP@(**1**) series. A striking deviation observed in the AgNP@(**2**) series compared with the AgNP@(**1**) series is the weaker dependency on their formation as a function of the [Tollens]. For example, a 1–50 mM concentration range of Tollens and a 100 μM – 25 mM concentration range of (**2**) is well-tolerated in AgNP@(**2**), which suggests that the structural differences in (**2**) relative to (**1**) plays an influential role in the size and morphology of the AgNP@(**2**). Indeed both the central resorcinol ether core as well as the three triazole units was critical for the preparation of AgNP@(**2**) as demonstrated by the formation of silver aggregates using (**4**) under the same conditions.

The influence of the density of reducing sugars on both AgNP size and shape control became apparent when the sugar triazole dendrimer comprising four reducing sugars (**3**) was utilized. In this series, formation of spherical and crystalline (fcc) AgNP@(**3**) of dimensions 9.7 ± 1.9 nm were formed (Figures 2c, S7-9). This is a similar size regime to that of the AgNP@(**2**) albeit with considerable improvements in monodispersity. Intriguingly, AgNP@(**3**) were formed over a similar concentration range of Tollens to the AgNP@(**2**) series, which suggests that AgNP formation using ligands (**2**) and (**3**) proceeds via a similar reaction mechanism. The trend of increase size and shape control of AgNP formation as a function of the number of reducing sugars [i.e. AgNP@(**3**) ≈ AgNP@(**2**) > AgNP@(**1**)] is particularly striking as these results suggest the capacity to control size and shape of AgNP growth by tuning both the number of reducing sugars as well as the nature of the central dendrimer core. This trend was also translated in their stability in buffered solutions. For example, AgNP@(**1**) remained dispersed in aqueous solutions containing up to 30 mM NaCl (Figure S2e in the SI), whereas AgNP@(**2**) and AgNP@(**3**) samples remained dispersed up to 30 mM and 2.8 M NaCl respectively at 20 °C over a period of 24 h (Figure S5e and S8e in the SI).

**Figure 3:**
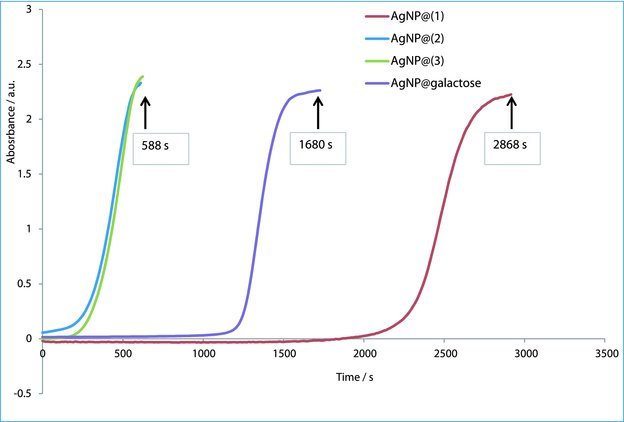
Kinetics of AgNP formation using sugar ligands (1, red), (2, blue), (3, green) and D-galactose (purple). AgNP formation was monitored by the formation of the surface Plasmon peak at 400 nm at the temperature of 20 °C.

Kinetics experiments were then conducted as a function of reducing sugar type (**1–3**) using the formation of the surface plasmon peak at ∼400 nm as a diagnostic marker of the formation of AgNPs. For each of the sugar triazoles (**1–3**) investigated, an autocatalytic process was observed (**Figure 3**). The onset of AgNP@(**1**) formation was observed at ∼2000 s with an endpoint at 2868 s using the optimized conditions for their formation. A significantly faster reaction rate [onset at ∼1000 s] was observed when D-galactose was used as the silver(I) reductant to form AgNP@D-galactose,4b suggesting that the triazole unit in (**1**) slows the rate of AgNP@(**1**) formation. In contrast to the AgNP@(**1**) and AgNP@D-galactose systems, the reaction kinetics of both AgNP@(**2**) and AgNP@(**3**) were significantly faster (Figure 3). Intriguingly, the rate of onset (∼120 s) and the end point (∼588 s) of both AgNP@(**2**) and AgNP@(**3**) were virtually identical.4b Thus, based on the kinetic data, an increase in the number of reducing sugars from one (**1**) to two (**2**) increases the rate of AgNP formation, however a further increase in the number of reducing sugars from two (**2**) to four (**3**) has little effect on the reaction rate. We therefore conclude that the presence of the triazole unit in sugar (**1**) slows the rate of AgNP@(**1**) formation relative to the rate of formation of AgNP@D-galactose. The rate of both AgNP@(**1**) and AgNP@D-galactose formation is significantly slower than AgNP@(**2**) and AgNP@(**3**), resulting in the following trend: rate of AgNP formation AgNP(**2**) ≈AgNP(**3**) ≫AgNP@D-galactose>AgNP@(**1**). This trend cannot be rationalized by an increase in only the reducing sugar moieties as the sugar (**3**) system should exhibit a considerably faster rate relative to sugar (**2**). We therefore conclude that the resorcinol ether core structure of the sugar triazole (**2**) is a key determinant in the increased rate of the reaction kinetics relative to (**1**).

The capacity of the central resorcinol ether core to mediate the nucleation and growth of AgNPs was intriguing as such a role has not previously been reported in the literature and initiated us to further probe how:

1. Ag^+^ interacts with sugar (**1**) and how this differs in the case of sugar (**2**); and
2. these differences could influence AgNP size and dispersity in solution at defined silver(I): (**1**)/(**2**) ratios.

^1^H NMR titrations were conducted in order to investigate these phenomena.

**Figure 4:**
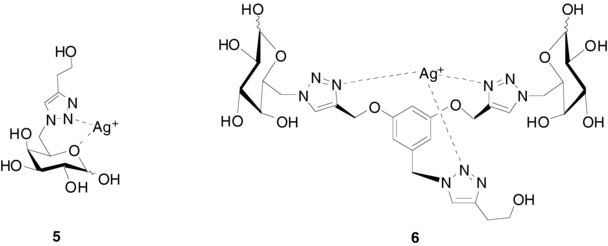
Proposed binding of Ag^+^ with (1) and (2).

The triazole proton (H_a_, Figure 1) and the anomeric sugar H1’ (H_b_, Figure 1) of (**1**) were used as diagnostic markers for Ag^+^ coordination. Titration of up to 1.0 equivalent of AgNO_3_ resulted in a 0.2 ppm downfield shift of the triazole proton H_a_ using a concentration of 4.2 mM of (**1**). This downfield shift reached a maximum at a 1: 1 Ag^+^: (**1**) ratio (Figure S10a and S14 in the SI). Concomitantly, a downfield shift (13 Hz, 0.026 ppm) of H_b_ was also observed, which, much akin to H_a_, reached a maximum at a 1: 1 Ag^+^: (**1**) stoichiometry, suggesting the formation of a Ag^+^·(**1**) complex. Collectively these results point towards the formation of a complex such as (**5**) (**Figure 4**), which involves Ag^+^ coordination to N2 of the triazole ring and the pyranosyl oxygen. We infer coordination to the pyranosyl oxygen because this would lead to the 6-membered ring chelate but we cannot rule out coordination to the anomeric hydroxyl group, although this is less likely as this would lead to a thermodynamically less stable 8-membered ring chelate. A comparable titration of Ag^+^ into an aqueous solution containing D-galactose did not afford a similar downfield shift of any of the sugar protons (Figure S10a, S17), highlighting the importance of the triazole unit in directing the coordination of Ag^+^ towards the pyranosyl oxygen.

Surprisingly, very different behavior was observed in the titration experiments when sugar (**2**) was used. In this example, triazole protons show a similar downfield response [i.e., 0.2 ppm or 100 Hz for H_c_ and 110 Hz for H_d_] to the addition of one equivalent of Ag^+^ at a concentration of 2 mM. The downfield shift of H_d_ then reaches a maximum at a Ag^+^: (**2**) ratio of 1: 1, whereas the downfield shift of H_c_ reaches maximum at a 2:1 Ag^+^: (**2**) ratio (Figure S10b and S15 in the SI). This suggests that in the presence of one equivalent of Ag^+^, all three triazole units work in concert to coordinate a single Ag^+^ cation, resulting in the putative formation of a novel tridentate complex (**6**). Consistent with the ability of the resorcinol ether core structure to provide an appropriate molecular framework to template the coordination of Ag^+^, similar Ag^+^-binding behavior was also observed with sugar (**4**) (Figure S18 in the SI), where in this respect, a concerted downfield shift of H_g_ protons was also observed. A significant deviation from the behavior observed in the Ag^+^•(**1**) complex is the lack of a downfield shift of the anomeric (H1’) proton in Ag^+^•(**2**) complexes, which is indicative of a different coordinating behavior and provides further support for the chelation of the Ag^+^ by the triazoles rather than the utilization of the pyranosyl oxygen. This is also underpinned by an *upfield* shift observed for both H_e_ and H_f_ up to a 1:1 Ag^+^: (**2**) ratio. An upfield shift of this kind has recently been observed in other triazole•Ag^+^ complexes, in which the authors suggest the presence of face-to-face π–π stacking interactions.^9^ Beyond a 1:1 Ag^+^: (**2**) stoichiometry results in a gradual return to a similar chemical shift position of H_e_ and H_f_ observed in the free ligand state after the addition of an excess amount of Ag^+^ (i.e., >3.0 equivalents). Taken collectively, these observations therefore suggest that sugar (**2**) coordinates one equivalent of Ag^+^ via the three triazoles, resulting in the formation of a species such as (**6**). Subsequent addition of Ag^+^ results in the formation of multimetal metallocyclic species.9a

Electrospray mass spectrometry (ES-MS) analysis of a Ag^+^·(**2**) complex at a 1:1 Ag^+^: (**2**) ratio afforded the molecular ion *m/z* 828 (Figure S20a in the SI) corresponding to the monomeric complex [(**2**) + Ag]^+^ as the predominant species present, whereas in the presence 3:1 Ag^+^: (**2**) ratios, molecular ions corresponding to [(**2**) + 2Ag]^+^ and [(**2**) + 3Ag]^+^ begin to form over [(**2**) + Ag]^+^ (Figure S20d and 20f). We therefore conclude that in Ag^+^·(**1**) complexes, Ag^+^ is coordinated to (**1**) in a 1:1 ratio via the triazole and most likely the pyranosyl oxygen to form a stable six-membered ring as in (**5**). In contrast, a tridendate system is formed when sugar (**2**) is used at 1:1 Ag^+^: (**2**) ratios, which crucially, does not involve coordination with the pyranosyl oxygen but in fact coordination of (**2**) with Ag^+^ is achieved via the three triazoles.

**Figure 5:**
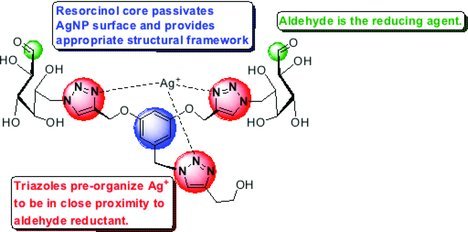
Proposed model for the formation of AgNPs using the template (2).

We then investigated the coordination properties of sugars (**1–3**) in the presence of the Tollens reagent in order to ascertain whether these deviations in Ag(I) coordination using AgNO_3_ and in sugars (**1**) and (**2**) also translated into different coordination behavior during AgNP formation. Using the triazole proton H_a_ as a probe, we observed a downfield shift from 7.82 ppm [free (**1**)] to 7.89 ppm after 10 minutes incubation with 1.0 equivalent of Tollens reagent (Figure S11 in the SI). This is only a minor downfield shift relative to the much larger chemical shift of 0.14 (57 Hz) ppm of H_a_ after the addition of 1.0 equivalent of AgNO_3_. As the formation of AgNP proceeds (monitored by surface plasmon formation) over a period of 24 h, an upfield shift of H_a_ to 7.86 was observed. When 2.0 equivalents of the Tollens reagent were added to sugar (**2**), the triazole peaks sharpened and split into the three distinct singlets after 10 min incubation time (Figure S12 in the SI). The most striking difference however was the formation of a sharp singlet at 8.80 ppm corresponding to an aldehyde proton. Under these conditions AgNP@(**2**) formation was observed which provides evidence that these shifts correspond to the ligand most likely bound to the surface and containing a residual aldehyde (Figure S15b in the SI). The same trend was also observed when sugar (**3**) was used. (Figure S13 and S16 in the SI). On the basis of our results, a general mechanistic framework describing how AgNP formation is mediated using (**2**) is proposed in **Figure 5**. According to ^1^H NMR titrations, the downfield shifts of H_a_ and H_b_ correspond to Ag^+^ coordination to a triazole nitrogen—most likely the nitrogen in the 2-position—and the pyranosyl oxygen to form a complex such as (**6**).

We rationalize this model as follows: Triazoles are well known to coordinate to a variety of transition metals, including Ag^+^.^9^, ^10^ The slower kinetics of AgNP@(**1**) formation relative to AgNP@D-galactose suggests that the coordination of the Ag^+^ to the pyranosyl oxygen in complex (**5**) hinders ring opening of the pyranose form of the D-galactose. Consequently the equilibrium is shifted more to the pyranose form, which in turn reduces the amount of aldehyde (the reductant) present, subsequently slowing down the rate of AgNP@(**1**) formation. Both AgNP@(**1**) and AgNP@D-galactose produce AgNPs in the similar size regime despite their different reaction kinetics, which infers that, in these systems, the initial rate of Ag(I) reduction might not be the rate limiting step, but rather the downstream rates of nucleation and growth of the AgNP process are the controlling factors. The moderate size and shape control is also evident in these systems and is consistent with previous findings by Panacek et al.4b and Kvitek et al.,4d which also suggests that the presence of the triazole in (**1**) has little influence on size control. In contrast to AgNPs produced using (**1**) and D-galactose, AgNPs formed using either sugar (**2**) or (**3**) as the Ag(I) reductant results in significant faster rates of AgNP formation. In these systems, the increase in the rate of AgNP formation correlates with AgNPs of much smaller size than those produced using either (**1**) or D-galactose. Surprisingly, there is little difference in size regimes of AgNP@(**2**) and AgNP@(**3**) as well as their rate of formation, which we surmise that the similar ligand cores of (**2**) and (**3**) relative to (**1**) is playing an influential and multifaceted role in AgNP formation. According to ^1^H NMR studies the ability to form AgNPs is directly associated with the unique capacity of the core of (**2**) and (**3**) to bind Ag(I) via a fundamentally different mechanism compared to (**1**). Both sugars (**2**) and (**3**) contain the same resorcinol-ether triazole core, which pre-assembles a Ag(I) cation in close proximity to the sites of reduction i.e. the D-galactose aldehyde. In 2006, Panigrahi et al. reported the preparation of AgNPs using resorcinol as a capping agent;^11^ however to the best of our knowledge, there have been no reports highlighting the utility of resorcinol ethers facilitating the controlled syntheses of AgNPs. Under our reaction conditions the open chain form of the D-galactose units in (**2**) presents two aldehyde functionalities in close proximity to a Ag(I) cation held in place by three triazole units as putatively highlighted in (**6**). This tridentate system is critical for size-controlled formation of AgNPs as no AgNPs are formed using (**4**) as the Ag(I) reductant. These results therefore highlight that the pre-complexation of Ag^+^ within an appropriate tridentate ligand complex can be utilized for the preparation of size and shape-controlled AgNPs of significantly smaller size regimes than that of 40 nm as previously reported using readily available mono-and disaccharides produced.4b,d,e

Despite similar reaction kinetics there are significant differences between AgNPs formed in the presence of (**2**) and (**3**). AgNPs derived from the use of ligand (**3**) exhibit far superior size and shape-control and remarkable colloidal stability in high-salt aqueous buffers relative to AgNP@(**2**). Although the molecular underpinnings of this are not completely understood at this point, we infer that the multiple resorcinol ether functionalities appear to facilitate not only size and shape control but also impart a vastly improved ability to passivate the AgNP surface. Panigrahi et al. reported a multifunctional role of resorcinol in the formation of highly stable AgNP suspensions in water albeit via a different mechanism to the one reported in this manuscript.^11^ In this respect, the authors inferred that the improved water stability of the AgNPs were as a consequence of the OH groups of resorcinol forming a superlattice structure on the AgNP surface. Although there are no phenolic OH groups present in our ligands, it is feasible that the corresponding pendent sugars of (**2**) and (**3**) could also form a unique superlattice structure on the AgNP surface. In this respect the higher sugar density present in (**3**) would therefore provide a more extensive and stable superlattice network, thereby explaining the unique stability of AgNP@(**3**) in high salt aqueous buffers relative to AgNP@(**2**). The molecular underpinnings of this phenomenon are currently being pursued in our laboratory and will be reported in due course.

In conclusion, this manuscript reports a novel synthetic strategy for the one-step synthesis of highly stable, size and shape-controlled AgNPs using sugar triazole ligands (**1–3**). The mild methods outlined in this manuscript provide opportunities to utilize derivatives of (**1–3**) outfitted with biomolecules such as nucleic acids and peptides to produce AgNPs in a single synthetic step. The availability of various derivatisation points in the central core of (**2**) enables tuneable and downstream derivatisation thus opening up new biomedical and material science-related opportunities for AgNPs where high stability in aqueous buffered solutions and tuneable size control are critical parameters.

## Supporting Information

Supporting Information is available from the Wiley Online Library or from the author. These include procedures for the preparation of sugar triazoles (**1–4**) and their use in the preparation of AgNP@(**1**), AgNP@(**2**), and AgNP@(**3**); characterization data of AgNP@(**1**), AgNP@(**2**), and AgNP@(**3**) using HR-TEM, DLS, and EDX; characterization data [^1^H NMR titration and ES-MS] of Ag^+^ complexes with (**1**), (**2**), and D-galactose; and characterization data [^1^H NMR titration] of [Ag(NH_3_)_2_]^+^ interactions with (**1**), (**2**), and (**3**).

## Supplementary material

Detailed facts of importance to specialist readers are published as ”Supporting Information”. Such documents are peer-reviewed, but not copy-edited or typeset. They are made available as submitted by the authors.

## References

[1a] Fan JA, Wu CH, Bao K, Bao JM, Bardhan R, Halas NJ, Manoharan VN, Nordlander P, Shvets G, Capasso F (2010). Science.

[b] Giljohann DA, Seferos DS, Daniel WL, Massich MD, Patel PC, Mirkin CA (2010). Angew. Chem. Int. Ed.

[c] Tan SJ, Campolongo MJ, Luo D, Cheng WL (2011). Nat. Nanotech.

[d] Liu SQ, Tang ZY (2010). J. Mat. Chem.

[e] Lu XM, Rycenga M, Skrabalak SE, Wiley B, Xia YN (2009). Ann. Rev. Phys. Chem.

[f] Grzelczak M, Perez-Juste J, Mulvaney P, Liz-Marzan LM (2008). Chem. Soc. Rev.

[g] Willets KA, Van Duyne RP (2007). Ann. Rev. Phys. Chem.

[2a] Brazier JA, Shibata T, Townsley J, Taylor BF, Frary E, Williams NH, Williams DM (2005). Nucleic Acids Res.

[b] Davis ME, Chen Z, Shin DM (2008). Nature Rev. Drug Discov.

[3a] García-Barrasa J, López-de-Luzuriaga J, Monge M (2011). Cent. Eur. J. Chem.

[b] Nair LS, Laurencin CT (2007). J. Biomed. Nanotech.

[c] Evanoff DD, Chumanov G (2005). ChemPhysChem.

[d] Larmour IA, Faulds K, Graham D (2010). J. Phys. Chem. C.

[e] MacAskill A, Crawford D, Graham D, Faulds K (2009). Anal. Chem.

[f] Graham D, Thompson DG, Smith WE, Faulds K (2008). Nat. Nanotech.

[4a] Lundahl P, Stokes R, Smith E, Martin R, Graham D (2008). Micro & Nano Lett. IET.

[b] Panáček A, Kvítek L, Prucek R, Kolář M, Večeřová R, Pizúrová N, Sharma VK, Nevěčná Tj, Zbořil R (2006). J. Phys. Chem. B.

[c] Qu L, Dai L (2005). J. Phys. Chem. B.

[d] Kvitek L, Prucek R, Panacek A, Novotny R, Hrbac J, Zboril R (2005). J. Mater. Chem.

[e] Yin Y, Li Z-Y, Zhong Z, Gates B, Xia Y, Venkateswaran S (2002). J. Mater. Chem.

[f] Belser K, Vig Slenters T, Pfumbidzai C, Upert G, Mirolo L, Fromm KM, Wennemers H (2009). Angew. Chem. Int. Ed.

[5a] Le A-T, Huy P, Huy T, Cam P, Kudrinskiy A, Olenin A, Lisichkin G, Krutyakov Y (2010). Nanotech. Russia.

[b] Soukupová J, Kvítek L, Panácek A, Nevecná T, Zboril R (2008). Mater. Chem. Phys.

[c] Kvitek L, Panaček A, Soukupova J, Kolar M, Večerova R, Prucek R, Holecova M, Zboril R (2008). J. Phys. Chem. C.

[d] Yu D, Yam VW-W (2005). J. Phys. Chem. B.

[e] Yu D, Yam VW-W (2004). J. Am. Chem. Soc.

[6] Gierlich J, Gutsmiedl K, Gramlich PME, Schmidt A, Burley GA, Carell T (2007). Chem.-Eur. J.

[7] Burley GA, Gierlich J, Mofid MR, Nir H, Tal S, Eichen Y, Carell T (2006). J. Am. Chem. Soc.

[8] Wu P, Feldman AK, Nugent AK, Hawker CJ, Scheel A, Voit B, Pyun J, Fréchet JMJ, Sharpless KB, Fokin VV (2004). Angew. Chem. Int. Ed.

[9a] Gower ML, Crowley JD (2010). Dalton Trans.

[b] Crowley JD, Bandeen PH, Hanton LR (2010). Polyhedron.

[c] Crowley JD, Bandeen PH (2010). Dalton Trans.

[10] Pergolese B, Muniz-Miranda M, Bigotto A (2004). J. Phys. Chem. B.

[11] Panigrahi S, Praharaj S, Basu S, Ghosh SK, Jana S, Pande S, Vo-Dinh T, Jiang H, Pal T (2006). J. Phys. Chem. B.

