# Supplementary material for: Highly Size-and Shape-Controlled Synthesis of Silver Nanoparticles via a Templated Tollens Reaction
Source: Small. 2012 Jan 9;8(5):770–6. doi: 10.1002/smll.201101474 (PMC3818698; doi:10.1002/smll.201101474)
Supplement: Supplementary file 1 [file smll0008-0770-sd1.pdf]

Copyright WILEY-VCH Verlag GmbH & Co. KGaA, 69469 Weinheim, Germany, 2012.

NANO small MICRO

Supporting Information

for *Small*, DOI: 10.1002/smll. 201101474

Highly Size- and Shape-Controlled Synthesis of Silver Nanoparticles via a Templated Tollens Reaction

Ruggero Dondi , Wu Su , Gerry A. Griffith , Graham Clark , and Glenn A. Burley *

Submitted to small NANO MICRO

Highly size and shape-controlled synthesis of silver nanoparticles via a templated Tollens reaction

*Ruggero Dondi,[1] Wu Su,[1] Gerry A. Griffith,[1] Graeme Clark[2] & Glenn A. Burley[3]**

[1]Department of Chemistry, University of Leicester
University Road, Leicester, LE1 1RE (UK).
[2] Department of Engineering, University of Leicester
University Road, Leicester, LE1 1RE (UK).
[*] [3]Department of Pure and Applied Chemistry, University of Strathclyde, Glasgow, G1 1XL (UK)
Email: glenn.burley@strath.ac.uk Webpage: www.burleylabs.co.uk

Table of Contents

1.0 Abbreviations 2
2.0 Experimental Section 2
2.1 General. 2
2.2 Synthesis of sugar control S3 2
2.3 Synthesis of sugar triazole (1) 5
2.4 Synthesis of sugar triazole (2) 5
2.5 Synthesis of sugar triazole (3) 6
3.0 Silver Nanoparticle (AgNP) formation 7
3.1 Preparation of AgNP@(1) series 8
4.0 Reaction kinetics of AgNP formation 25
5.0 NMR studies of Ag^+ coordination with sugars (1), D-galactose and (2) 26
NMR studies of AgNP formation 29
5.1 NMR studies of using (1) 31
5.2 NMR studies of using (2) 32
5.3 NMR studies of using (3) 33
5.4 NMR studies of using D-galactose 34
5.4 NMR studies using (4) 35
6.0 ES-MS analysis of (1) 35
6.1 ES-MS analysis of (2) 37
7.0 References 42

1.0 Abbreviations

HRTEM: High resolution transmission electron microscopy

TEM: Transmission electron microscopy

SAED: Selected Area Electron Diffraction

EDX: Energy-dispersive X-ray

DMSO: Dimethylsulphoxide

HPLC: High Pressure Liquid Chromatography

UPLC-TOF: Ultra-high pressure liquid chromatography-Time of Flight

TBTA: tris-(Benzyltriazolylmethyl)amine, a click-chemistry ligand.

2.0 Experimental Section

2.1 General.

Silver nitrate (99.9999%), NH_3 (28%) were bought from Sigma Aldrich. Sugars **S4**, **S5** and **S6** were prepared as reported previously.[1] UV-Vis measurement were taken with a Thermo-Scientific Nanodrop 1000. Time-course kinetics were registered with a Perkin Elmer Lambda 35 fitted with a PTP6+6, a Varian Cary 50 Probe and a Shimadzu UV-2401PC. TEM images were acquired with a Jeol JEM 1400 with an Olympus Megaview III camera, a Jeol JEM 2100 with a GatanUltrascan 1000 camera and a Jeol JEM 2100F with a GatanOrius SC1000-2 high speed camera. TEM images were subsequently edited with ImageJ software (http://imagej.nih.gov). Mass spectra were obtained with a Waters AquityXevo and aMicromass Quattro LC. DLS data measurements were taken with a Malvern Instruments Zetasizer Nano S. GC-MS spectra were taken with a Perkin Elmer Autosystem XL + Turbomass. Ultrapure water was generated by an Elgastat Option 4 water purifier.

2.2 Synthesis of sugar control S3

Preparation of **S1**: to a stirred solution of resorcinol (2.000 g, 18.2 mmol) in acetone (150 mL) was added propargyl bromide (80% in Toluene, 4.3 g, 36.3 mmol), 18-crown-6 (0.02 g, 0.07 mmol), anhydrous and freshly grinded potassium carbonate (2.760 g, 19.9 mmol) under a nitrogen atmosphere. The reaction mixture was stirred 24h at reflux followed by concentration in vacuo to a small residue. The crude residue was then diluted with DCM (100 mL) and water (100 mL). The organic layer was separated, washed with Brine (80 mL), dried with

$MgSO_4$ and concentrated to a small volume. The concentrated solution was then purified by column chromatography over Si-60 using 100% DCM as eluent. 3-(prop-2-yn-1-yloxy)phenol (0.417g, 17%) and **S1** (2.586 g, 77%) were recovered as a pale yellow oils. EIMS *m/z:* $[M^+]$ 186, ^{1}H NMR (300 MHz, $CDCl_3$, δ): 7.13 (1H, m, H_3), 6.54 (3H, m, H_2 H_4 H_6), 4.59 (4H, d, *J*=2.3Hz, H_9 H_{10}), 2.45 (2H, t, *J*=2.3Hz, H_{13} H_{14})

Scheme S1: Synthesis of compound S1

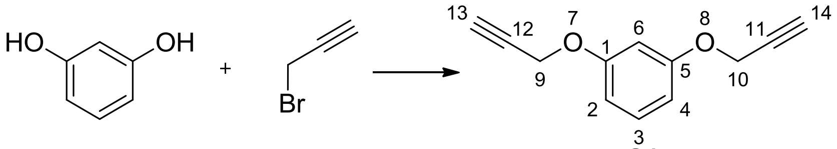

S1

Preparation of **S2**: to a stirred solution of **S1** (0.550 g, 2.96 mmol) in EtOH (10 mL) was added **S4** (0.551 g, 1.93 mmol), CuBr (0.061 g, 0.43 mmol), TBTA (0.190 g, 0.44 mmol) under a nitrogen atmosphere. The reaction mixture was stirred overnight at r.t. followed by concentration in vacuo, dilution with DCM (5 mL) and purification by column chromatography (Biotage 40L Si-60 column using Hexane and Acetate as solvents). Mono clicked product (0.384 g, 33%), unreacted **S1** (0.284 g, 52%) and **S2**(0.330 g, 15%)were recovered. ^{1}H NMR (300 MHz, $CDCl_3$, δ): 7.79 (2H, s, H_{12}), 7.20 (1H, m, H_2), 6.62 (3H, m, H_1 H_3 H_4), 5.52 (2H, d, *J*=5.4 Hz, H_6), 5.20 (4H, s, H_5), 4.62 (4H, m, H_7 H_{11}), 4.46 (2H, m, H_{10}), 4.33 (2H, m, H_8), 4.19 (4H, m, H_9), 1.49 (6H, s, CH_3isopropylidene), 1.39 (6H, s, CH_3isopropylidene), 1.36 (6H, s, CH_3isopropylidene), 1.30 (6H, s, CH_3isopropylidene)

Scheme S2: Synthesis of sugar S2

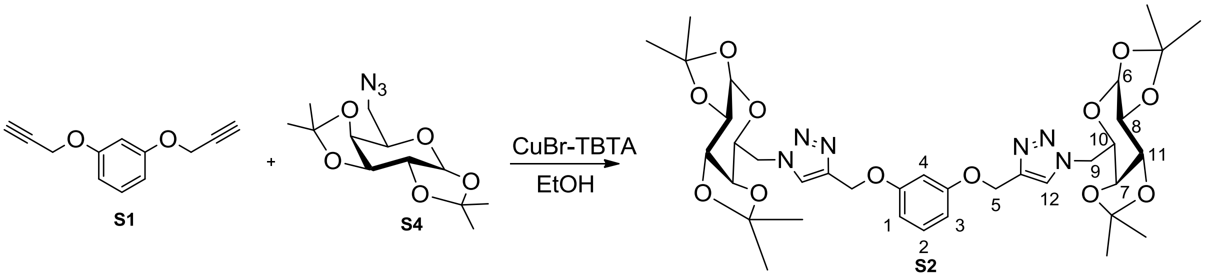

Preparation of **S3**: to a mixture of TFA : water (1 : 1, 10 mL) was added **S2** (0.330 g, 0.44mmol.) under a nitrogen atmosphere. The reaction mixture was heated to 65 °C and followed by TLC until complete (3 hours). The mixture was then cooled, concentrated in vacuo to a small volume, diluted with water (1.5 mL) and purified by semi-preparative HPLC. **S3** (55.2 mg, 21%) was recovered as a white powder after lyophilisation. HRMS (ESI) *m/z:* [M + H]$^+$ calcd 597.2156; found, 597.2164. ^{1}H NMR (300 MHz, $CDCl_3$, mixture of 4 diastereoisomers, δ): 8.18 (m, triazoles C-H), 7.21 (m, aromatic ring), 6.74 (m, aromatic ring), 6.65 (m, aromatic ring + sugars anomeric protons), 6.24 (m, sugars anomeric protons), 5.25 (m, sugars), 5.11 (m, sugars + CH_2), 4.94 (m, sugars), 4.75 (m, sugars), 4.50 (m, sugars), 4.27 (m, sugars), 3.91 (m, sugars), 3.72 (m, sugars), 3.57 (m, sugars)

Scheme S3: Synthesis of sugar S3. Selected area COSY-90 of sugar S3: red arrows highlight couplings of anomeric protons; the anomeric protons of the diastereoisomers show a difference of ~1 ppm in the chemical shift.

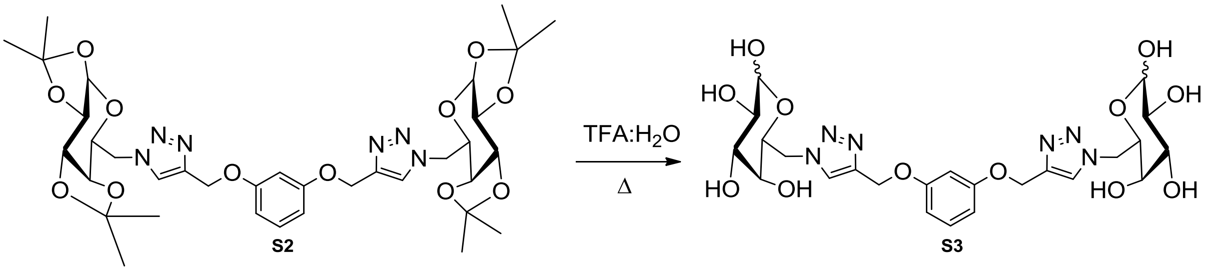

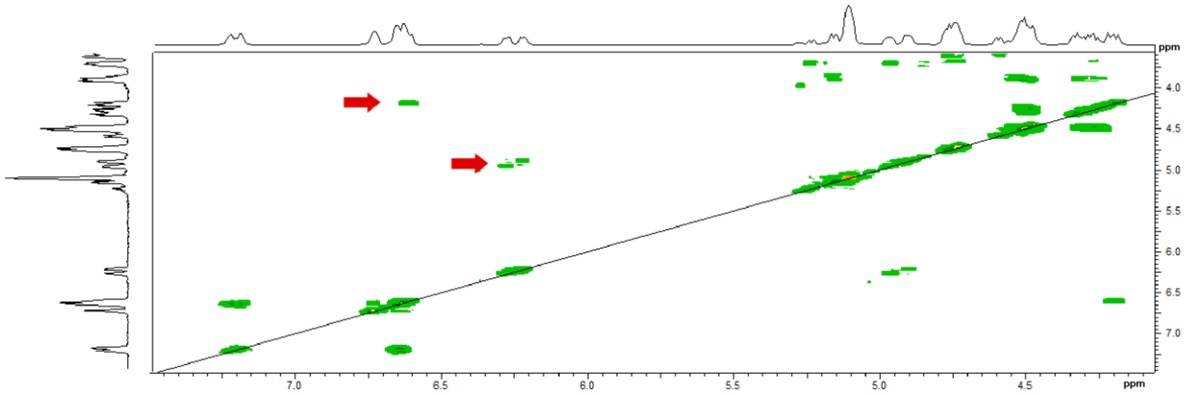

2.3 Synthesis of sugar triazole (1)

Preparation of **S4b**: to a stirred solution of **S4** (0.832 g, 2.9 mmol.) in DMSO (2 mL) was added Butyn-3-ol (0.205 g, 2.92 mmol.), and SiO_2-NHC-Cu(I)[2] (1.49 mmol/g catalyst loading, 2.150 g, 3.20 mmol) under a nitrogen atmosphere. The reaction mixture was stirred overnight followed by quenching with Brine (20 mL). The crude mixture was then filtered to remove the click-chemistry catalyst and extracted with ethyl acetate (3 x 20 mL). The combined organic layers were washed with brine (5 x 50 mL), 1.3% NH_3 (2 x 50 mL) and finally brine (50 mL). The organic layer was then dried (MgSO4), filtered and concentrated in vacuo to obtain **S4b** (0.693 g, 67%) as a pale yellow powder. HRMS (ESI) m/z: $[M + H]^+$ calc. 356.1822; found, 356.1817. ^{1}H NMR (300MHz ,$CDCl_3$, δ) = 7.50 (s, 1 H), 5.43 (d, 1 H), 4.62 - 4.44 (m, 2 H), 4.42 - 3.98 (m, 4 H), 3.96 - 3.75 (m, 2 H), 2.88 (s, 3 H), 1.49 - 1.11 (m, 12 H); ^{13}C NMR (100MHz ,$CDCl_3$, δ) =145.13, 122.85, 109.85, 109.04, 96.21, 71.17, 70.72, 70.31, 67.31, 61.71, 50.44, 28.72, 25.94, 25.88, 24.86, 24.39.

Preparation of **(1)**: to a mixture of TFA : water (1 : 1, 10 mL) was added **S4b** (0.693 g, 1.946mmol) under a nitrogen atmosphere. The reaction mixture was heated to 70 °C and was followed by TLC until complete. The mixture was then cooled, concentrated in vacuo to a small volume, diluted with water (10 mL) and lyophilized overnight to afford **(1)** (0.322 g, 60 %) as a white powder. HRMS (ESI) m/z: $[M + H]^+$ calcd 276.1196; found, 276.1193.

Scheme S4: Synthesis of sugar triazole (**1**)

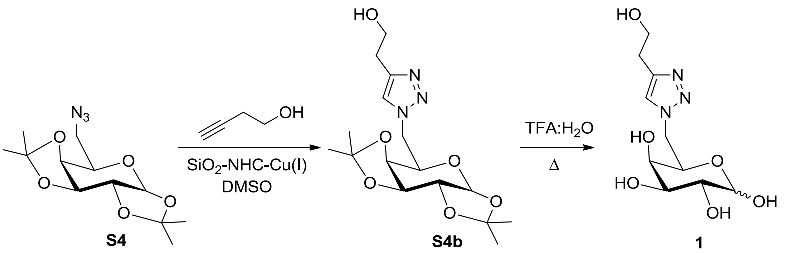

2.4 Synthesis of sugar triazole (2)

To a stirred solution of **S5** (0.303 g, 0.465 mmol.) in DMSO (10 mL) was added Butyn-3-ol (32mg, 0.465 mmol.), and SiO_2-NHC-Cu(I)[2] (1.49 mmol/g catalyst loading, 31 mg, 0.047 mmol) under a nitrogen atmosphere. The reaction mixture was stirred overnight then filtered with a 0.2 μm filter, the solid catalyst was washed with DMSO (1 mL) and the combined

filtrate was concentrated to a small volume, diluted with water (1.5 mL) and purified by semi-preparative HPLC. Lyophilisation of selected fractions afforded **(2)** (0.310 g, 91%) as a pale yellow powder. HRMS (ESI) *m/z:* $[M + H]^+$ calcd 722.2746; found, 722.2748.

Scheme S5: Synthesis of sugar triazole (**2**).

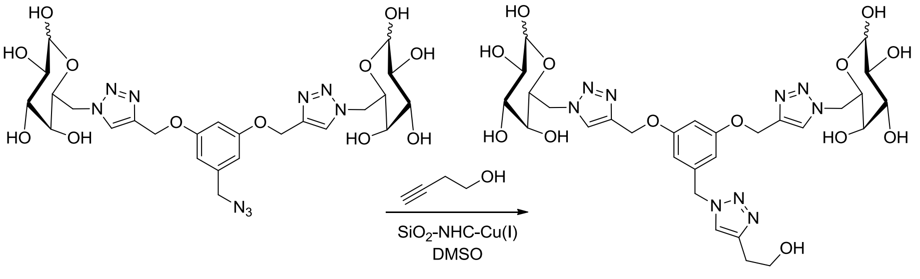

S5 2

2.5 Synthesis of sugar triazole (3)

To a stirred solution of **S6** (0.057 g, 0.037mmol.) in DMSO (3 mL) was added Butyn-3-ol (2.6mg, 0.037mmol.), and SiO_2-NHC-Cu(I) (1.49 mmol/g catalyst loading, 3.7 mg, 0.006mmol) under a nitrogen atmosphere. The reaction mixture was stirred overnight then filtered with a 0.2 μm filter, the solid catalyst was washed with DMSO (1 mL) and the combined filtrate was concentrated to a small volume, diluted with water (1.5 mL) and purified by semi-preparative HPLC.Lyophilisation of selected fractions afforded **(3)** (0.053 g, 92%) as a pale yellow powder. HRMS (ESI) m/z: $[M + H]^+$ calcd 1614.5846; found, 1614.5908.

Scheme S6: Synthesis of sugar triazole (**3**).

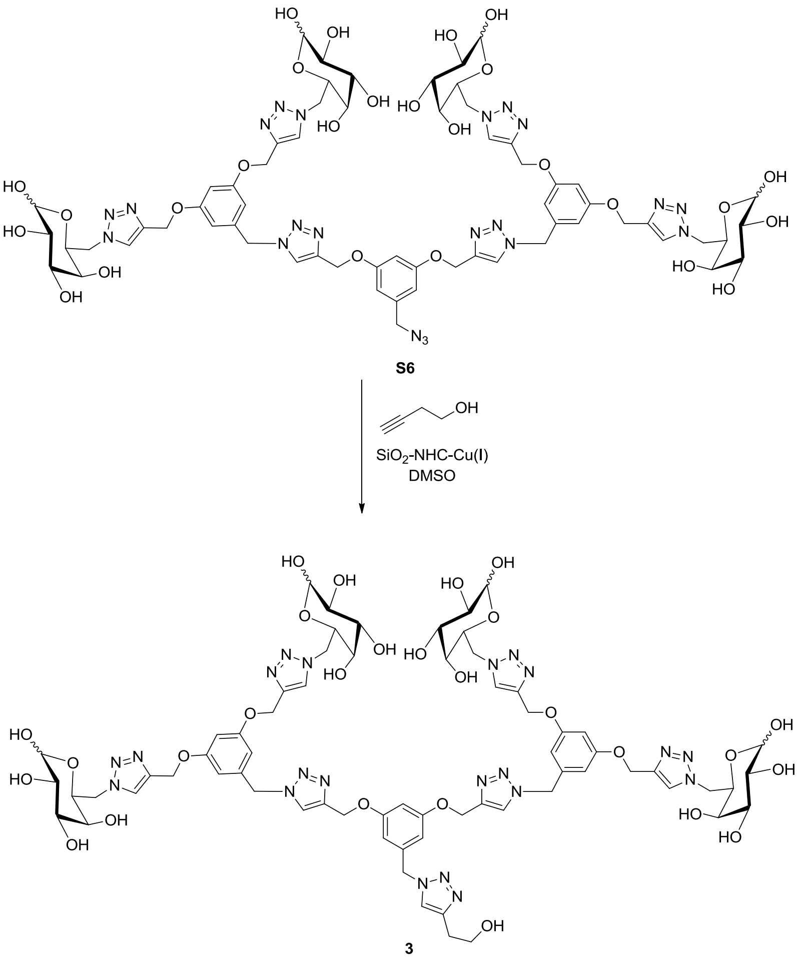

3.0 Silver Nanoparticle (AgNP) formation

Preparation of sugars stock solutions: sugars (1) and (2) were dissolved in ultrapure water and diluted as necessary with ultrapure water for the preparation of the arrays. Sugar (3) is relatively less soluble; the concentrated (10 mM) stock solution in ultrapure water was warmed at 40°C for 15 minutes prior to use. All the sugars stock solutions were preserved at 4°C.

Preparation of Tollens stock solutions: Tollens stock solutions were prepared fresh on a daily basis from 3 equivalent recipes and diluted as necessary for the nanoparticles arrays:

100 mMTollens: to 1.790 mL H_2O was added $AgNO_3$ (0.5M, 500 uL), followed by NaOH (3M, 100 ul) and finally NH_4OH (28%, 110 ul)

20 mMTollens: to 4059.2 mL H_2O was added $AgNO_3$ (0.5M, 278.4 uL), followed by NaOH (3M, 55.7 ul) and finally NH_4OH (28%, 61.25 ul)

3 mMTollens: to 9.915 mL H_2O was added $AgNO_3$ (0.5M, 60 uL), followed by NaOH (3M, 12 ul) and finally NH_4OH (28%, 13.2 ul)

General procedure for the preparation of AgNP: 300uL of Tollensstock solution and 300uL of sugar stock solution were mixed in disposable plastic Eppendorf. The solution was briefly shaken and left in the dark for 24 hours. The mixture was then centrifuged for 20 secs to remove occasional micrometer sized particles, then collection of surnatant afforded the AgNP colloid without any further purification.

3.1 Preparation of AgNP@(1) series

(a)

Sugar1

Tollens

	25mM	10mM	1mM	100uM	10uM	1uM
10uM	#1	#2	#3	#4	#5	#6
100uM	#7	#8	#9	#10	#11	#12
1mM	#13	#14	#15	#16	#17	#18
10mM	#19	#20	#21	#22	#23	#24
20mM	#25	#26	#27	#28	#29	#30
50mM	#31	#32	#33	#34	#35	#36

(b)

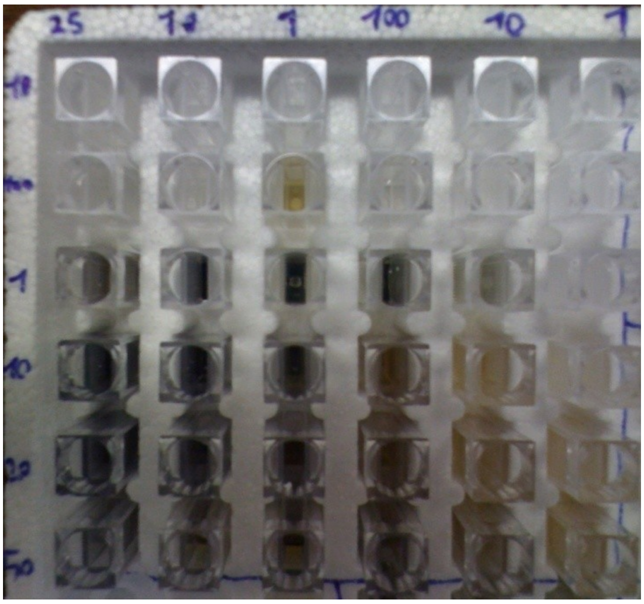

Figure S1: Array series for the preparation of AgNP@(**1**). **(a)** Schematic representation of the array used to investigate AgNP@(**1**) formation as a function of [Tollens] and [(**1**)]. **(b)** Photograph of the array to investigate AgNP@(**1**) formation as a function of [Tollens] and [(**1**)].

(a)

		Sugar1					
		25mM	10mM	1mM	100uM	10uM	1uM
Tollens	10uM	#1	#2	#3	#4	#5	#6
	100uM	#7	#8	#9	#10	#11	#12
	1mM	#13	#14	#15	#16	#17	#18
	10mM	#19	#20	#21	#22	#23	#24
	20mM	#25	#26	#27	#28	#29	#30
	50mM	#31	#32	#33	#34	#35	#36

(b)

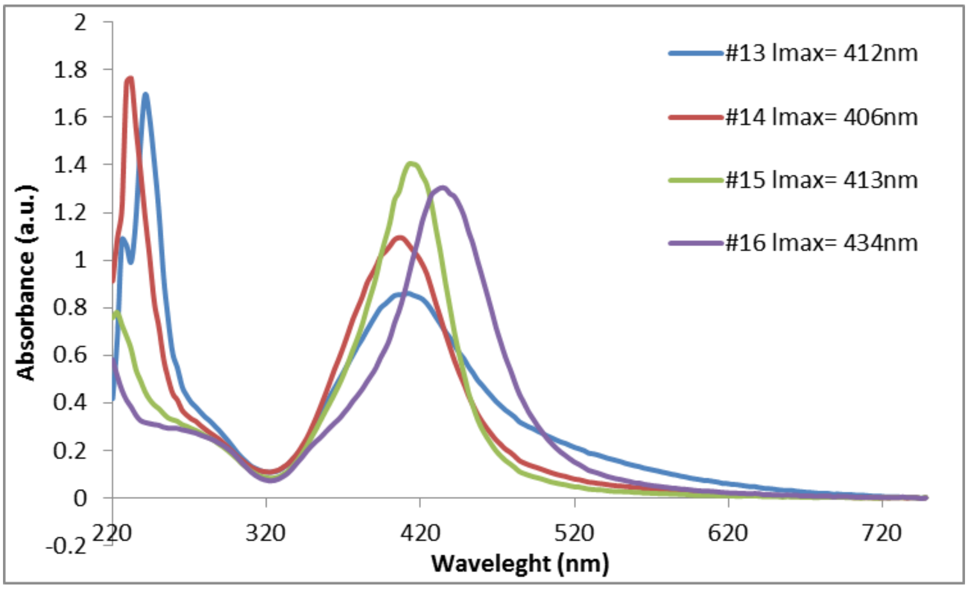
Absorbance (a.u.)
Waveleght (nm)
#13 lmax= 412nm
#14 lmax= 406nm
#15 lmax= 413nm
#16 lmax= 434nm
2
1.8
1.6
1.4
1.2
1
0.8
0.6
0.4
0.2
0
-0.2
220
320
420
520
620
720

(c)

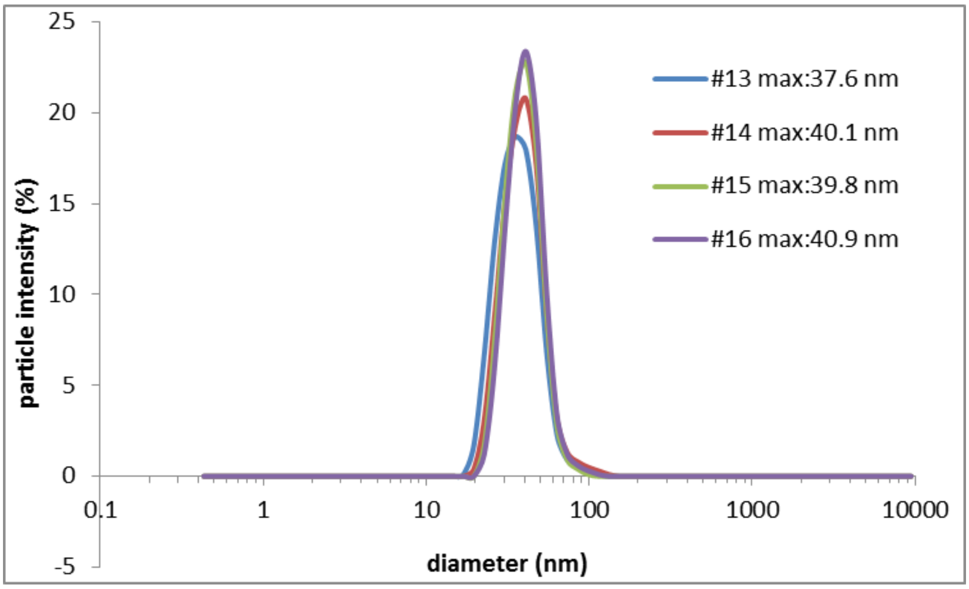
particle intensity (%)
diameter (nm)
#13 max:37.6 nm
#14 max:40.1 nm
#15 max:39.8 nm
#16 max:40.9 nm
25
20
15
10
5
0
-5
0.1
1
10
100
1000
10000

(d)

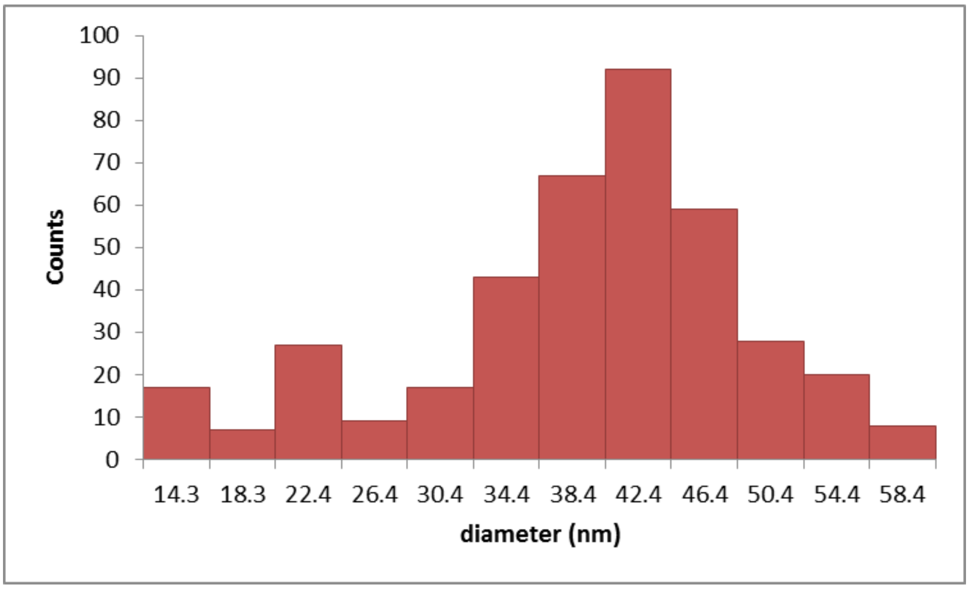

(e)

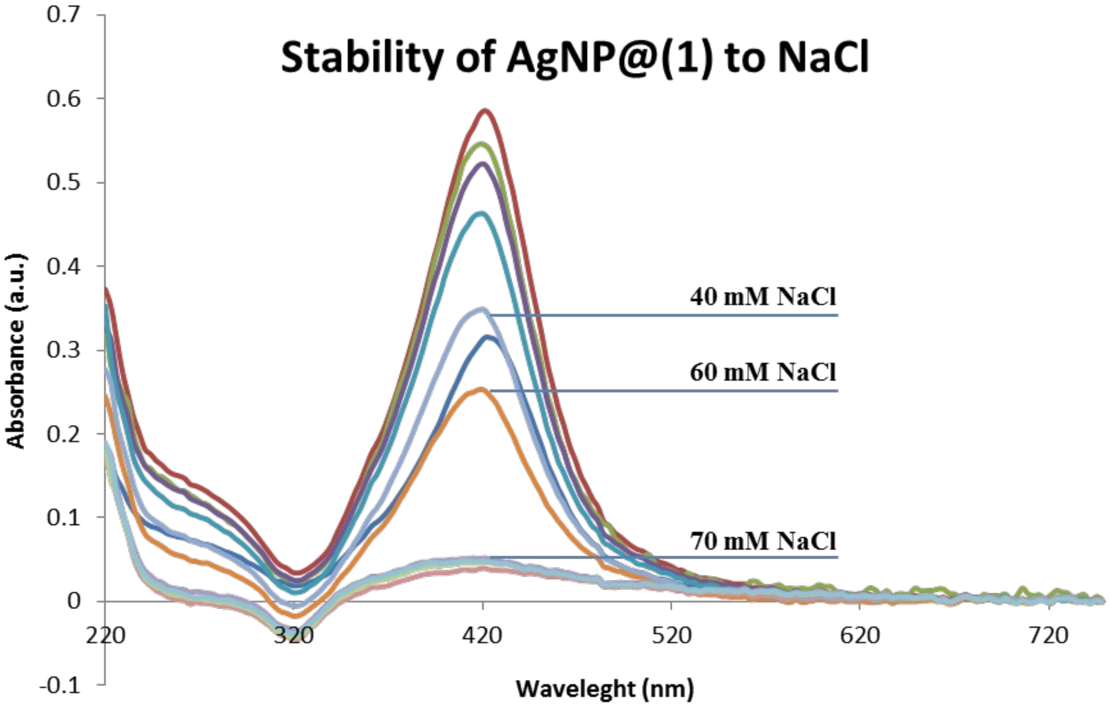

Figure S2: (a) Array series for the preparation of AgNP@(**1**). White boxes represent no AgNP formation, Yellow boxes represent AgNP formation and Grey boxes represent Ag aggregate (silver mirror) formation. **(b)** UV-vis spectra of reactions #13-16 which formed AgNPs as observed by a surface Plasmon peak. **(c)** Dynamic Light Scattering (DLS) of reactions #13-16 which formed AgNPs colloids. **(d)** Dispersity of sample #16 showing an average of 41.2 nm ± 25% (384 particles counted), values are in good agreement with the DLS data, **(e)** Stability of sample #16 to increasing concentrations of NaCl: UV-Vis of #16 in the presence of 0, 10, 20, 30, 40,

50, 60, 70, 80, 90, 100 mM NaCl. Spectra were registered after 24 hours incubation at 22°C. Particles start aggregating at 40mM NaCl and are completely aggregated at 70mM NaCl.

(a)

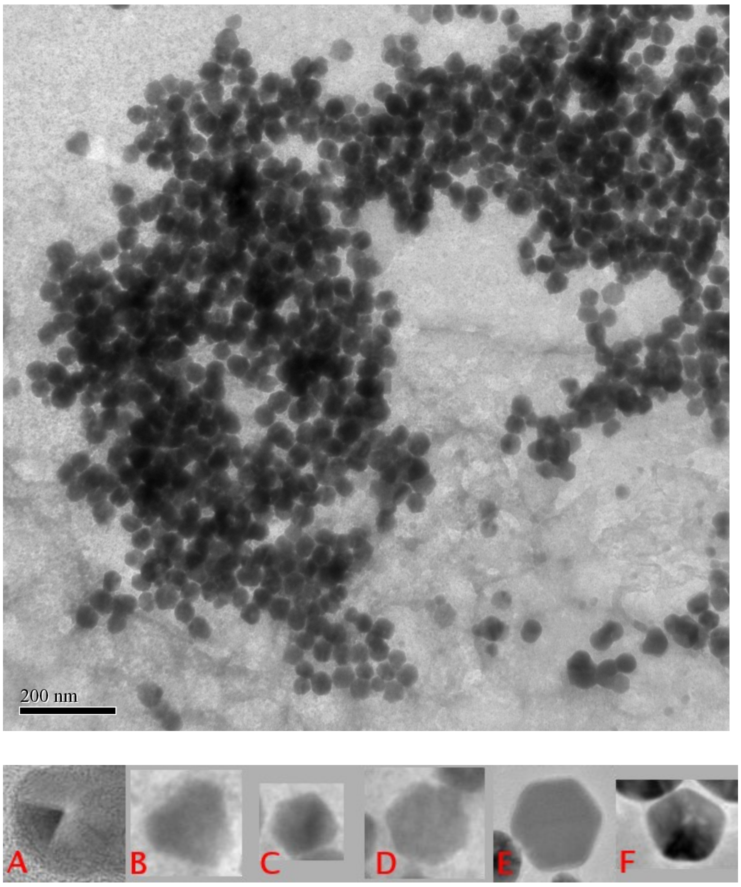

(b)

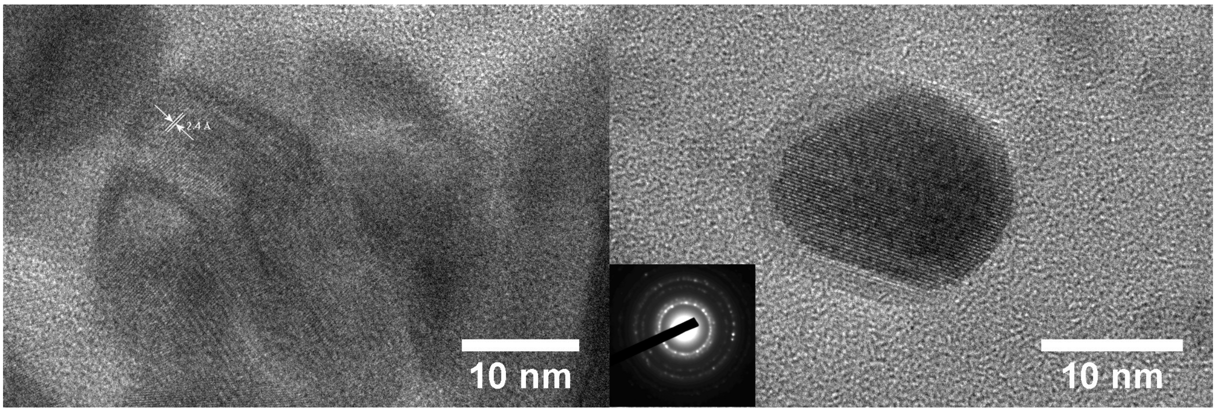

(c)

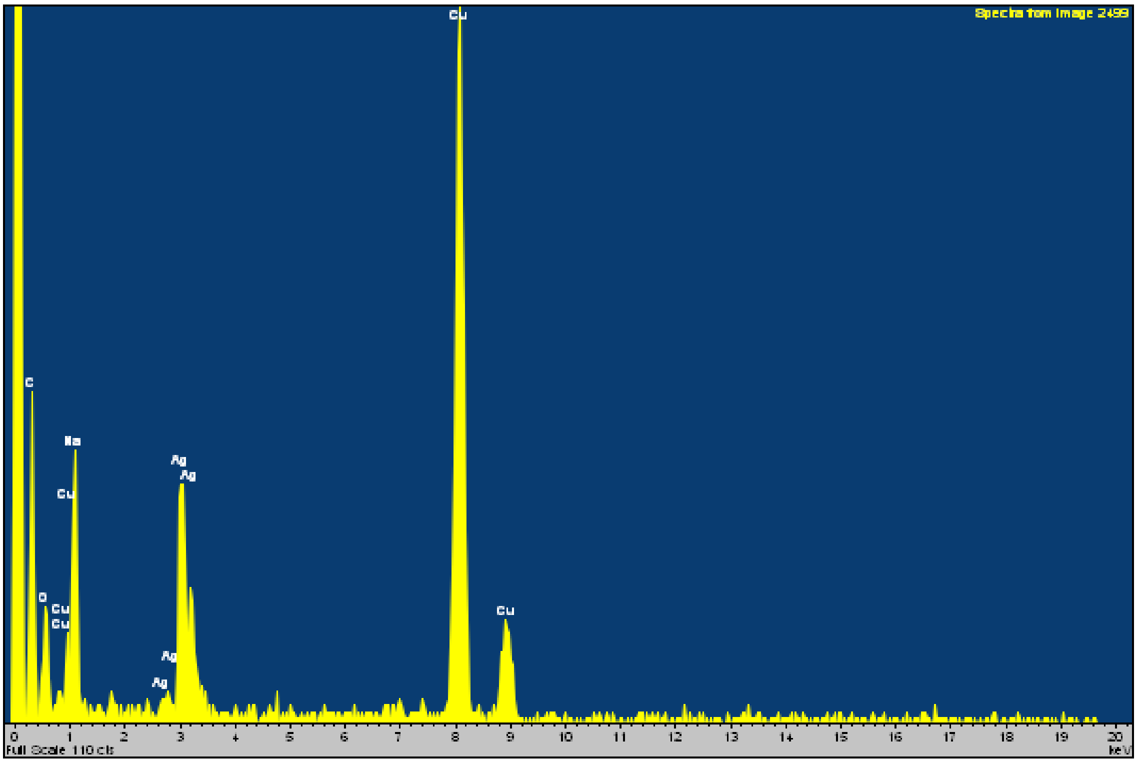

Figure S3: TEM images of AgNP prepared by the reduction of $[Ag(NH_3)_2]^+$ with (**1**) using 1 mM Tollens and 100 μM (**2**) [i.e. reaction condition #16 highlighted in Figure S2(a)]. **(a)** Selected polyhedra of AgNP@(**1**) formed using reaction condition #16: A. Rounded Icosahedron B. Truncated triangular plate C. Truncated

octahedron D/E. Hexagonal plate F. Icosahedron. **(b)** HR-TEM of a representative NP of the AgNP@(**1**) series derived from reaction condition #16. Fringe spacing of 2.4 Å can be assigned to {111} reflection of face cubic centered Ag. In the inset SAED confirms crystalline fcc Ag. **(c)** EDX of AgNP@(**1**) series derived from reaction condition #16.

3.2 Preparation of AgNP@(2) series

(a)

[Sugar2]

[Tollens]

	25mM	10mM	1mM	100uM	10uM
100uM	#1	#2	#3	#4	#5
1mM	#6	#7	#8	#9	#10
10mM	#11	#12	#13	#14	#15
20mM	#16	#17	#18	#19	#20
50mM	#21	#22	#23	#24	#25

(b)

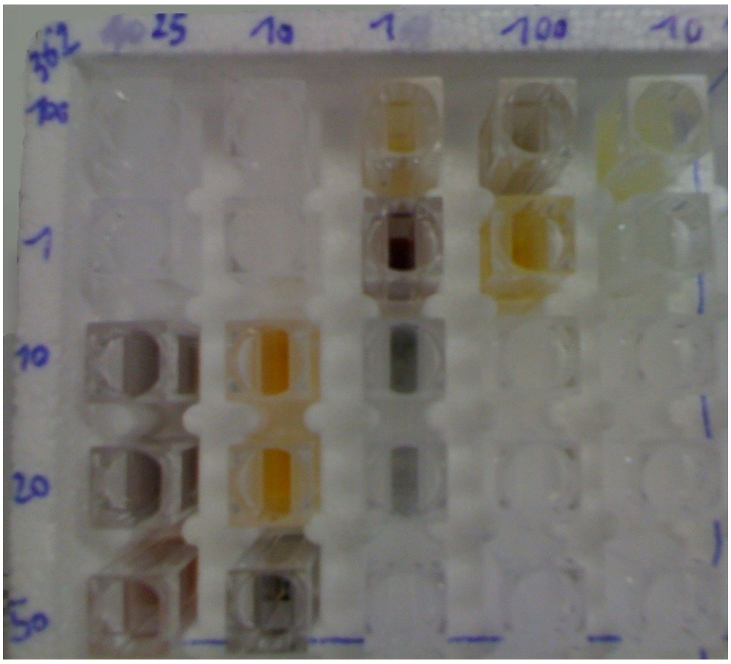

Figure S4: Array series for the preparation of AgNP@(**2**). **(a)** Schematic representation of the array used to investigate AgNP@(**2**) formation as a function of [Tollens] and [(**2**)]. **(b)** Photograph of the array to investigate AgNP@(**2**) formation as a function of [Tollens] and [(**2**)].

(a)

Sugar2 (columns); **Tollens** (rows)

	25mM	10mM	1mM	100uM	10uM
100uM	#1	#2	#3	#4	#5
1mM	#6	#7	#8	#9	#10
10mM	#11	#12	#13	#14	#15
20mM	#16	#17	#18	#19	#20
50mM	#21	#22	#23	#24	#25

(b)

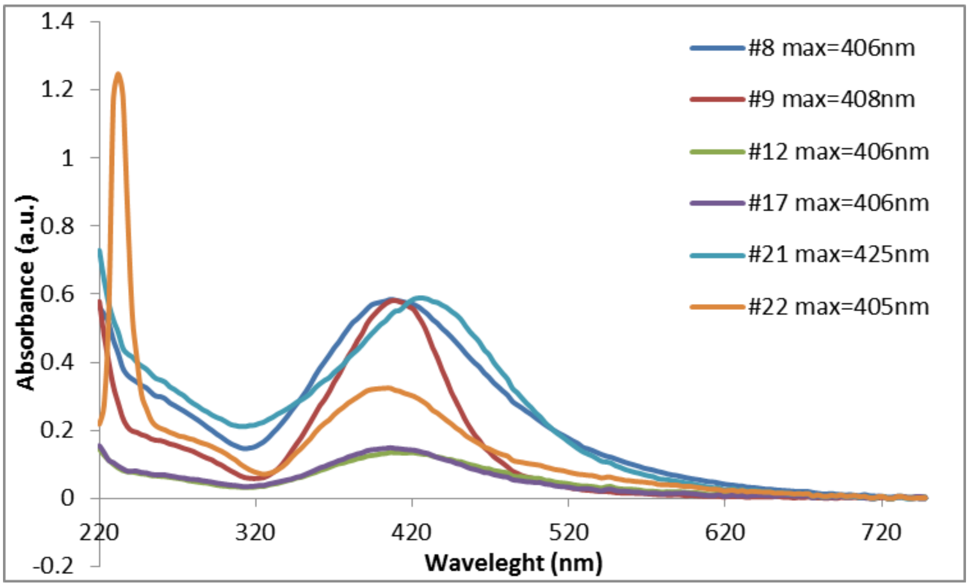
1.4
1.2
1
0.8
0.6
0.4
0.2
0
-0.2
Absorbance (a.u.)
220
320
420
520
620
720
Waveleght (nm)
#8 max=406nm
#9 max=408nm
#12 max=406nm
#17 max=406nm
#21 max=425nm
#22 max=405nm

(c)

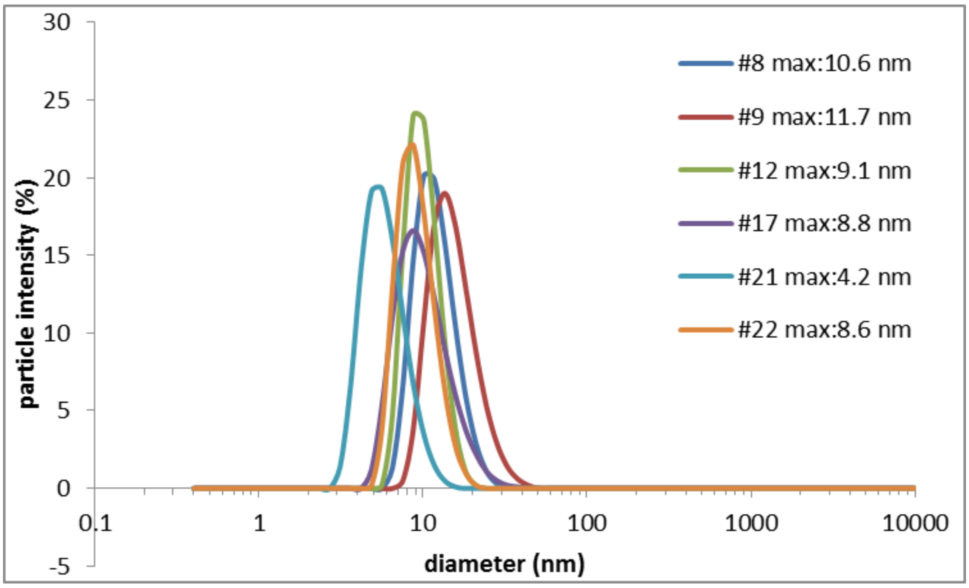
30
25
20
15
10
5
0
-5
particle intensity (%)
0.1
1
10
100
1000
10000
diameter (nm)
#8 max:10.6 nm
#9 max:11.7 nm
#12 max:9.1 nm
#17 max:8.8 nm
#21 max:4.2 nm
#22 max:8.6 nm

(d)

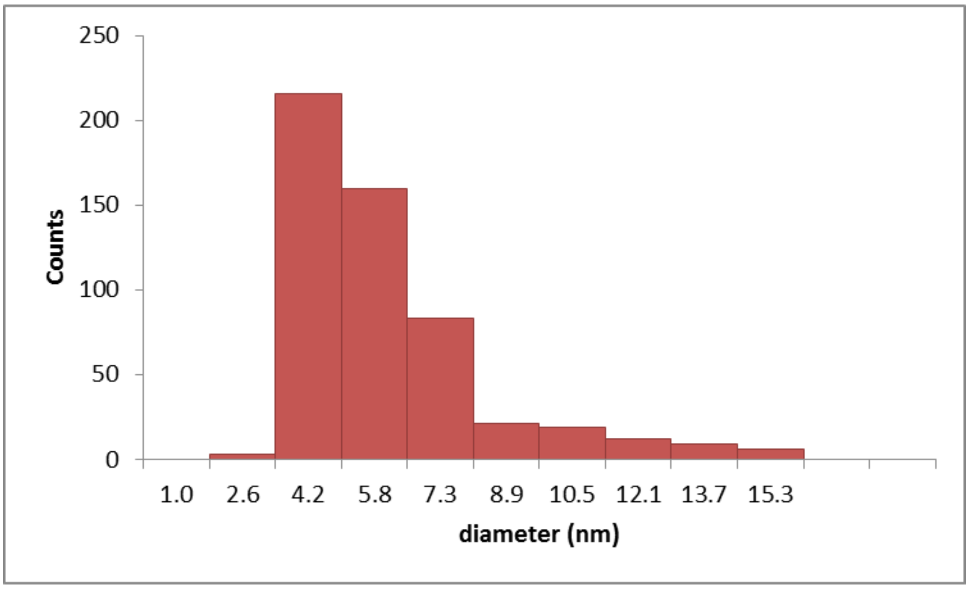

(e)

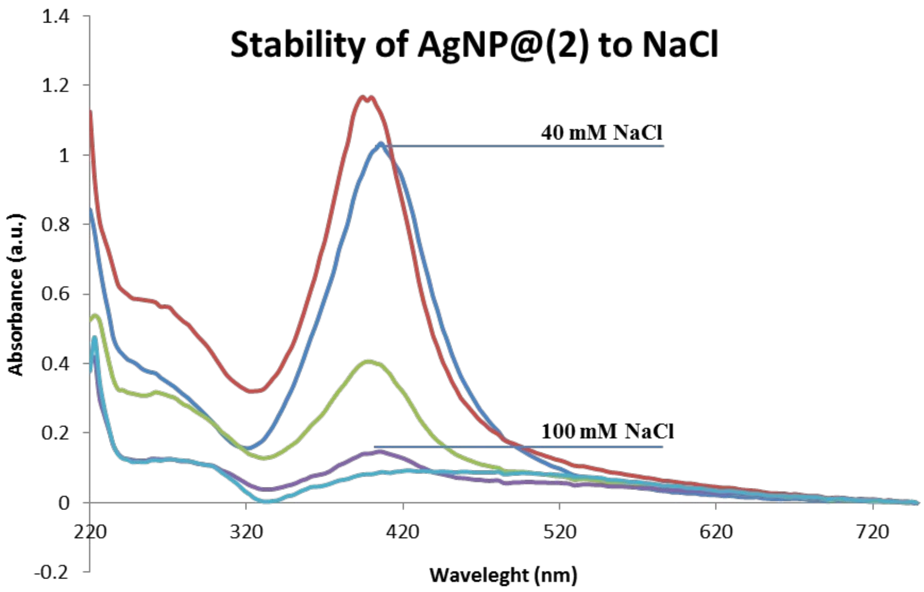

Figure S5: (a) Array series for the preparation of AgNP@(**2**). White boxes represent no AgNP formation, Yellow boxes represent AgNP formation. **(b)** UV-vis spectra of reactions #8-9,12,17,21-22 which formed AgNPs as observed by a surface Plasmon peak. Samples #17-21-22 were diluted 1:20 prior the measurements. **(c)** DLS of reactions #8-9,12,17,21-22 which formed AgNPs colloids. **(d)** Dispersity of sample #17 showing an average of 7.9 nm ± 62% (568 particles counted), values are in good agreement with the DLS data. **(e)** Stability of sample #17 to increasing concentrations of NaCl: UV-Vis of #17 in the presence of 0, 40, 60, 100, 120 mM NaCl. Spectra were registered after 24 hours incubation at 22°C. Particles start aggregating at 40mM NaCl and are completely aggregated at 100mM NaCl.

(a)

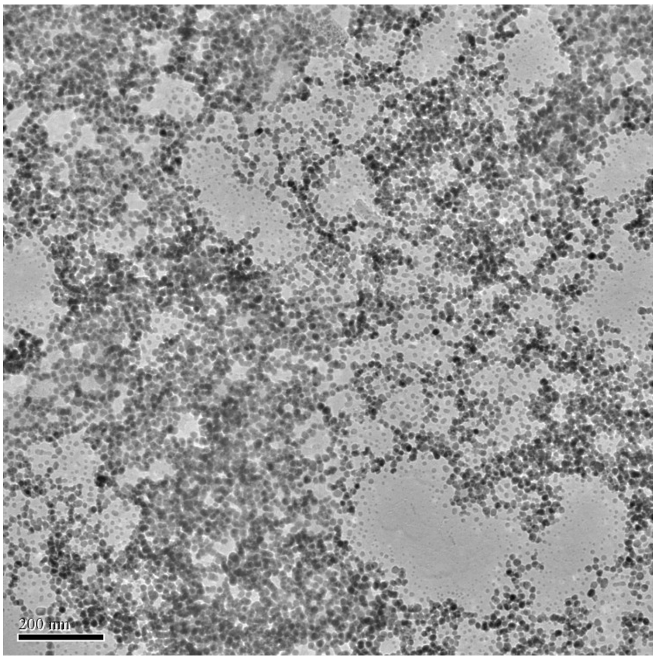

(b)

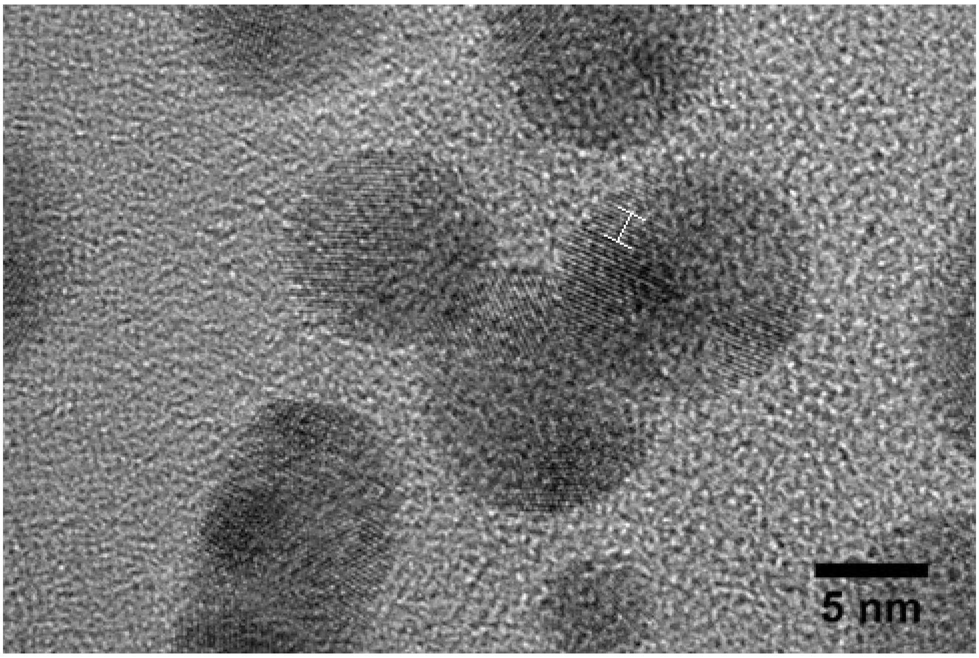

•

(c)

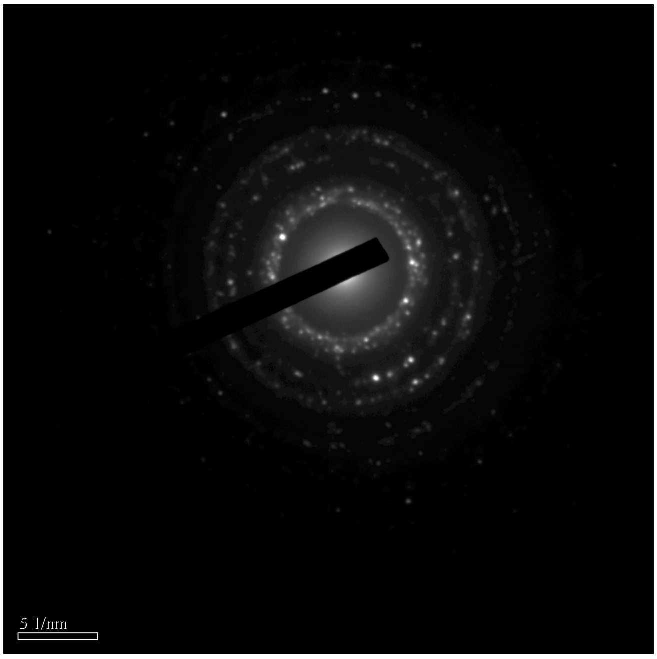
5 1/nm

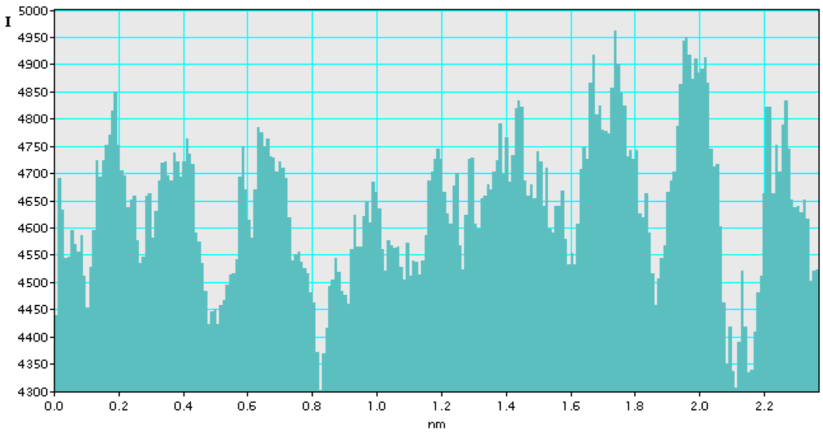
I
5000
4950
4900
4850
4800
4750
4700
4650
4600
4550
4500
4450
4400
4350
4300
0.0
0.2
0.4
0.6
0.8
1.0
1.2
1.4
1.6
1.8
2.0
2.2
nm

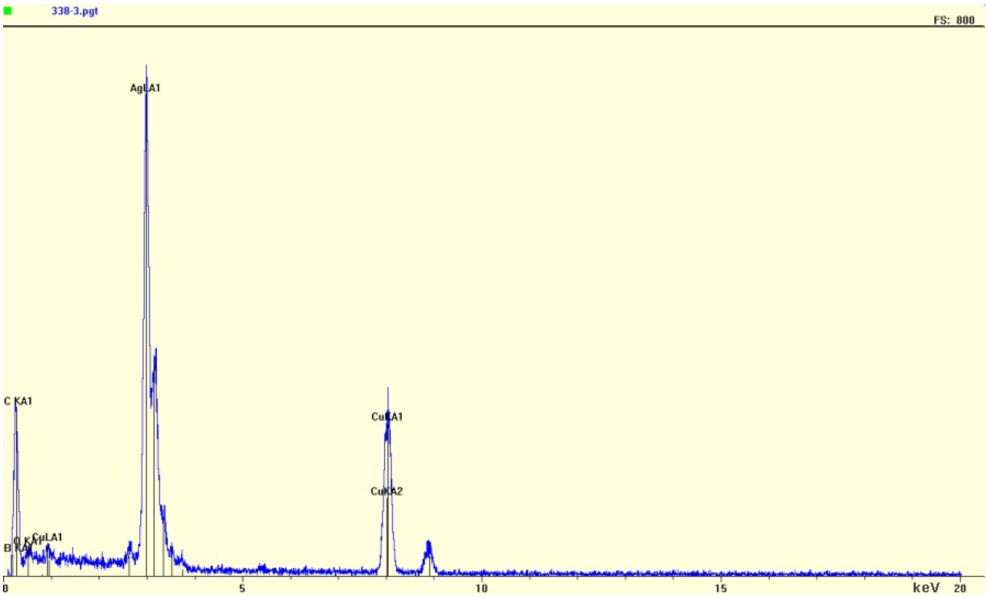

Figure S6: TEM images of AgNP prepared by the reduction of $[Ag(NH_3)_2]^+$ with (**2**) using 20 mM Tollens and 10μM (**2**) [i.e. reaction condition #17 highlighted in Figure S5(a)]. **(a)** TEM of the population of AgNP@(**2**) formed using reaction condition #17. **(b)** HR-TEM of selected AgNP@(**2**) formed using reaction condition #17. **(c)** SAED, fringing plot and EDX of AgNP@(**2**) particle highlighted in (b).

3.3 Preparation of AgNP@(3) series

(a)

[Sugar4]

[Tollens]

	5mM	1mM	100uM	10uM	1uM
100uM	#1	#2	#3	#4	#5
1mM	#6	#7	#8	#9	#10
10mM	#11	#12	#13	#14	#15
20mM	#16	#17	#18	#19	#20
50mM	#21	#22	#23	#24	#25

(b)

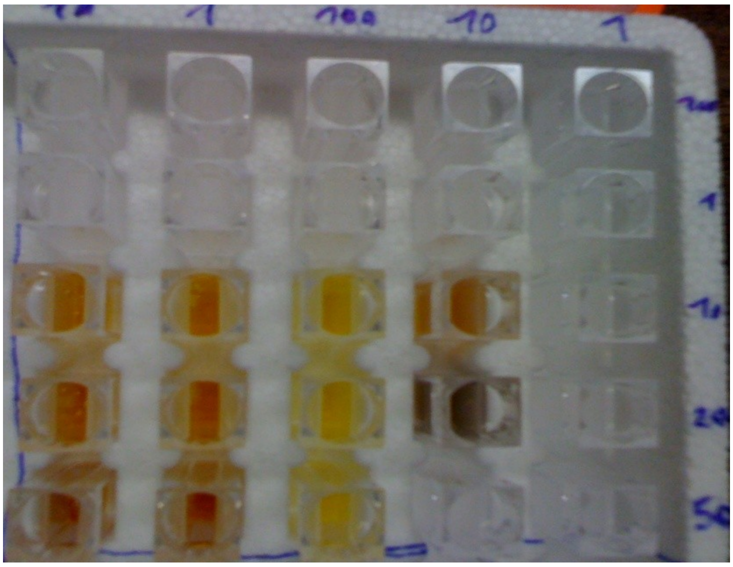

Figure S7: Array series for the preparation of AgNP@(**3**). **(a)** Schematic representation of the array used to investigate AgNP@(**2**) formation as a function of [Tollens] and [(**3**)]. **(b)** Photograph of the array to investigate AgNP@(**3**) formation as a function of [Tollens] and [(**3**)].

(a)

[Sugar]

[Tollens]

	5mM	1mM	100uM	10uM	1uM
100uM	#1	#2	#3	#4	#5
1mM	#6	#7	#8	#9	#10
10mM	#11	#12	#13	#14	#15
20mM	#16	#17	#18	#19	#20
50mM	#21	#22	#23	#24	#25

(b)

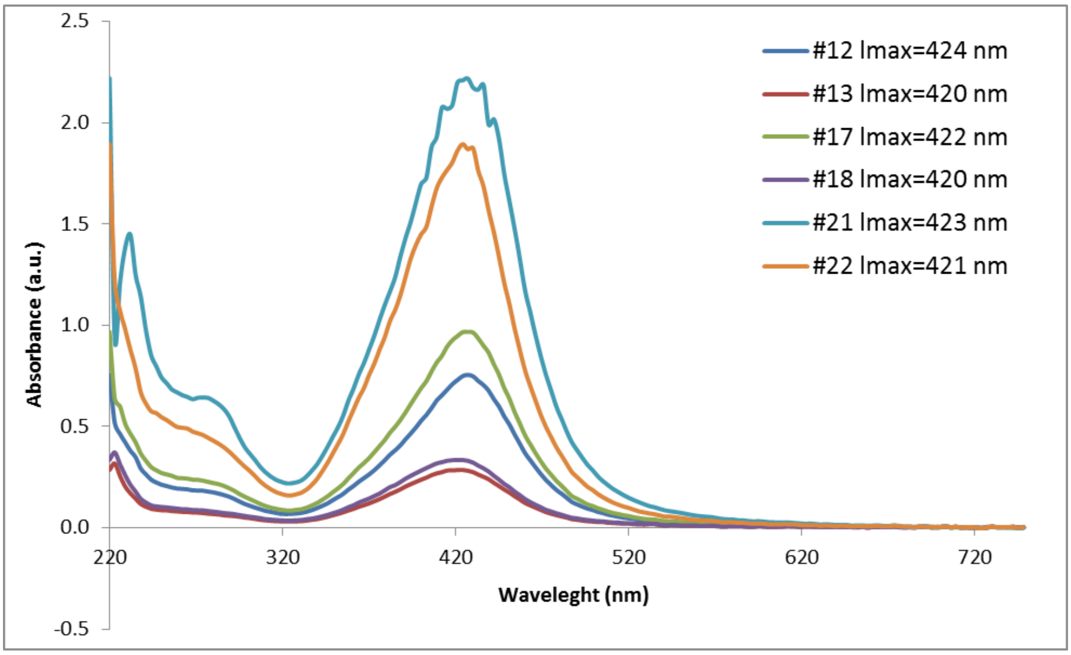
Absorbance (a.u.)
Waveleght (nm)
2.5
2.0
1.5
1.0
0.5
0.0
-0.5
220
320
420
520
620
720
#12 lmax=424 nm
#13 lmax=420 nm
#17 lmax=422 nm
#18 lmax=420 nm
#21 lmax=423 nm
#22 lmax=421 nm

(c)

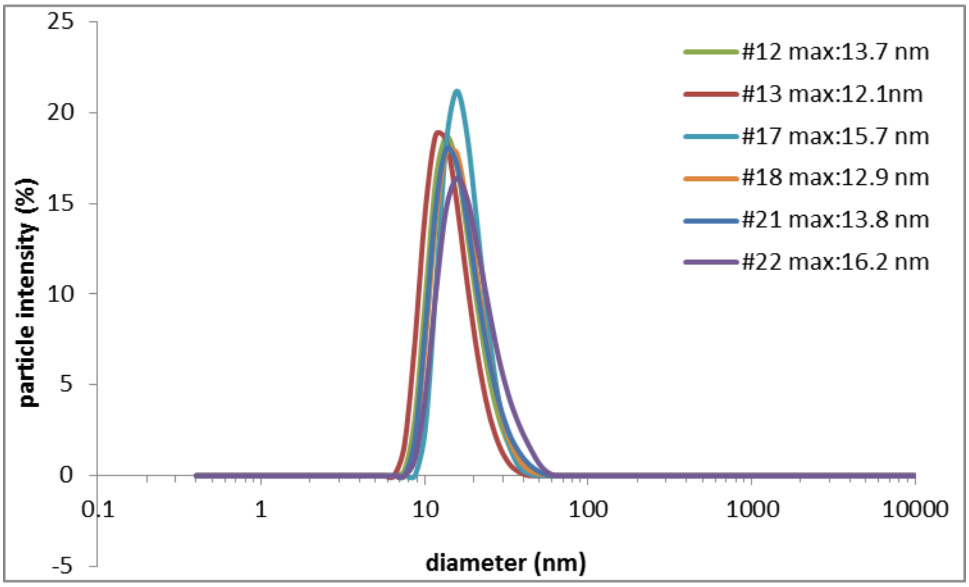
particle intensity (%)
diameter (nm)
25
20
15
10
5
0
-5
0.1
1
10
100
1000
10000
#12 max:13.7 nm
#13 max:12.1nm
#17 max:15.7 nm
#18 max:12.9 nm
#21 max:13.8 nm
#22 max:16.2 nm

(d)

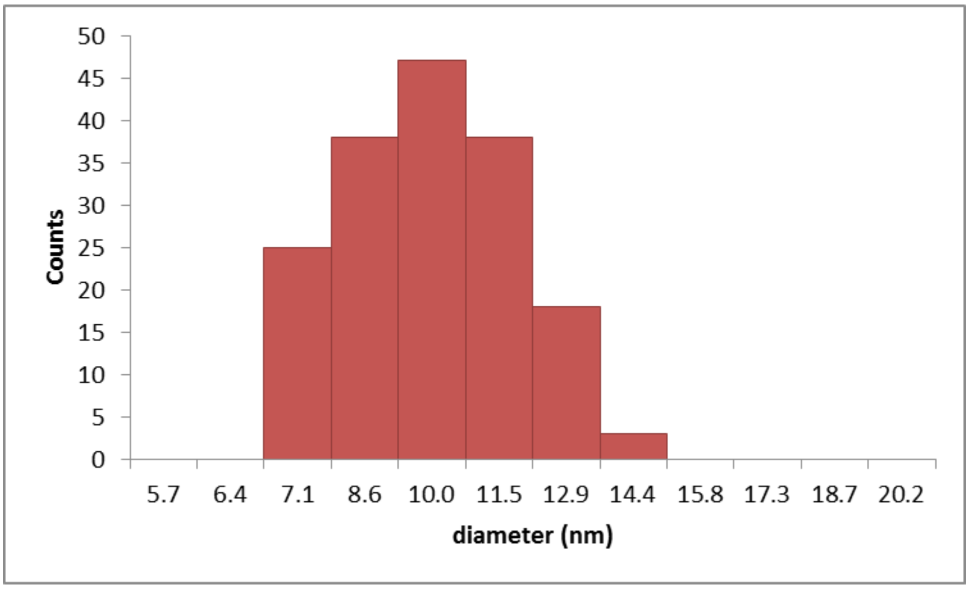

(e)

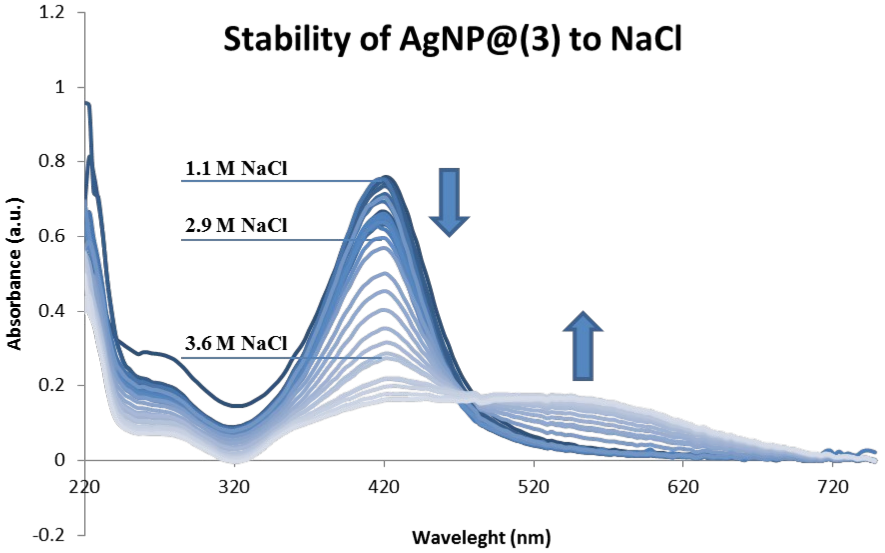

Figure S8: (a) Array series for the preparation of AgNP@(**2**). White boxes represent no AgNP formation, Yellow boxes represent AgNP formation. **(b)** UV-Vis spectra of reactions #12-13,17-18,21-22 which formed AgNPs as observed by a surface Plasmon peak. **(c)** DLS of reactions #12-13,17-18,21-22 which formed AgNPs as observed by a surface Plasmon peak. **(d)** Dispersity of sample #12 showing an average of 9.7 nm ± 20% (160 particles counted), values are in good agreement with the DLS data. **(e)** Stability of sample #12 to increasing concentrations of NaCl: UV-Vis of #12 in the presence of 0 M NaCl, 0.1 M NaCl, 1.0-4.2 M at 0.1 M NaCl intervals (35 spectra in total). Spectra were registered after 24 hours incubation at 22°C. Particles start aggregating at 2.9M NaCl and are completely aggregated at 3.6M NaCl

(a)

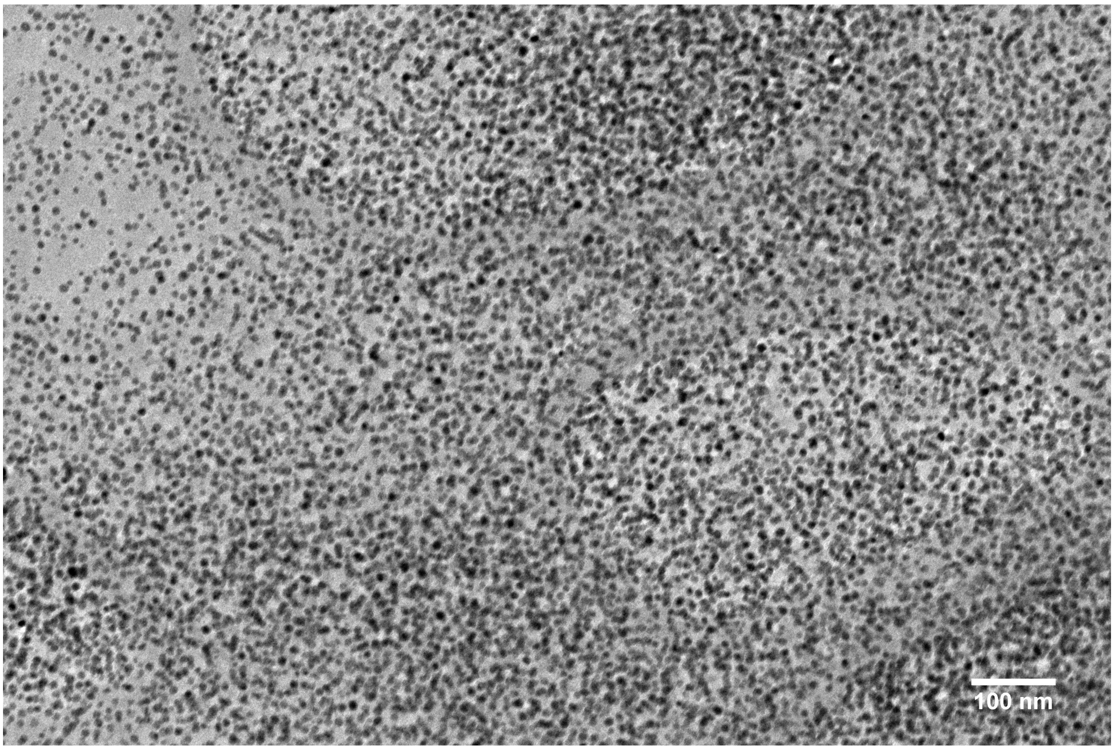

(b)

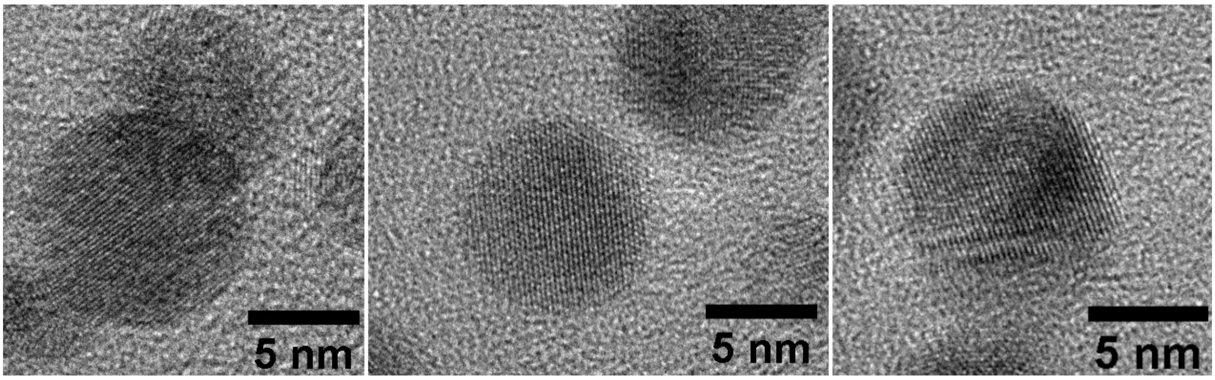

(c)

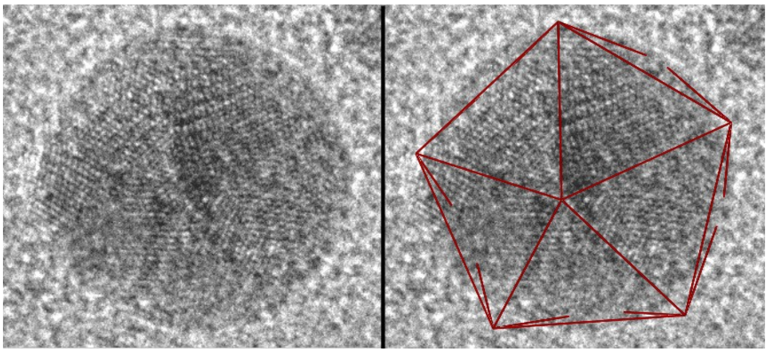

(d)

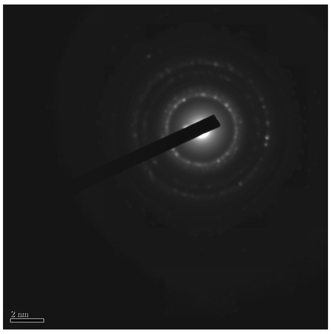

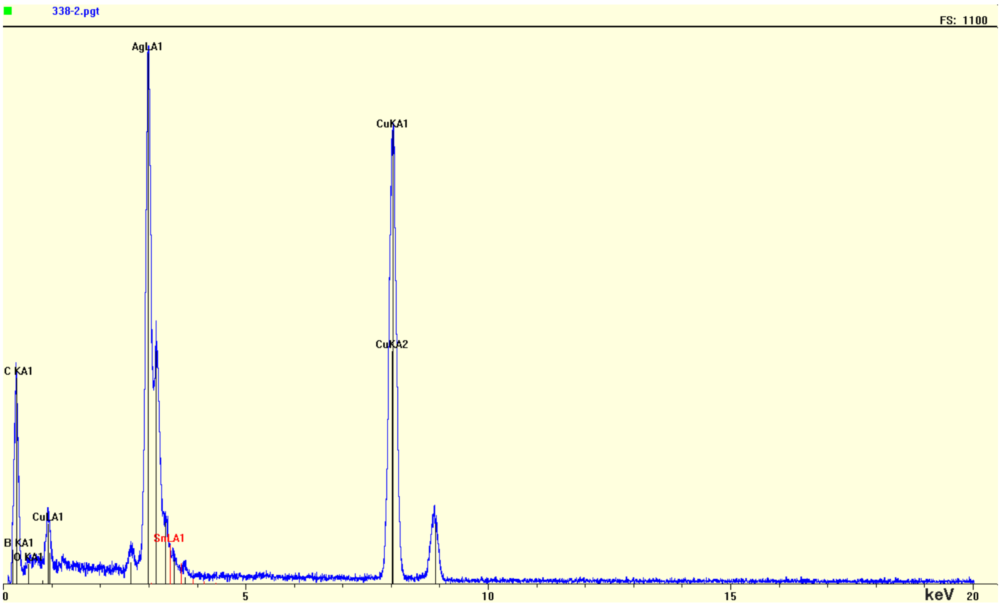

Figure S9: TEM images of AgNP prepared by the reduction of $[Ag(NH_3)_2]^+$ with (**3**) using 10mM Tollens and 1uM (**3**) [i.e. reaction condition #12 highlighted in Figure S8 (a)]. **(a)**TEM of the population of AgNP@(**3**) formed using reaction condition #12. **(b)** HR-TEM of selected AgNP@(**3**) formed using reaction condition #12. **(c)** Example of Icosahedral particle formed using reaction condition #12. On the right part of the image an overlay of the same particle with a wireframe regular icosahedron rotated to match the particle. **(d)** SAED and EDX of AgNP@(**3**) of the particle on the right part of (b).

4.0 Reaction kinetics of AgNP formation

Time course experiments protocol: 300 µL of sugar solution at 200 µM and 300 µL of Tollens solution at 2 mM were mixed in a low-volume quartz cuvette; UV-Vis measurement were taken at 400 nm every 5 seconds using a UV-Vis spectrophotometer fitted with a temperature-regulated cuvette holder set at 20°C.

5.0 NMR studies of Ag^{+} coordination with sugars (1), D-galactose and (2)

(a)

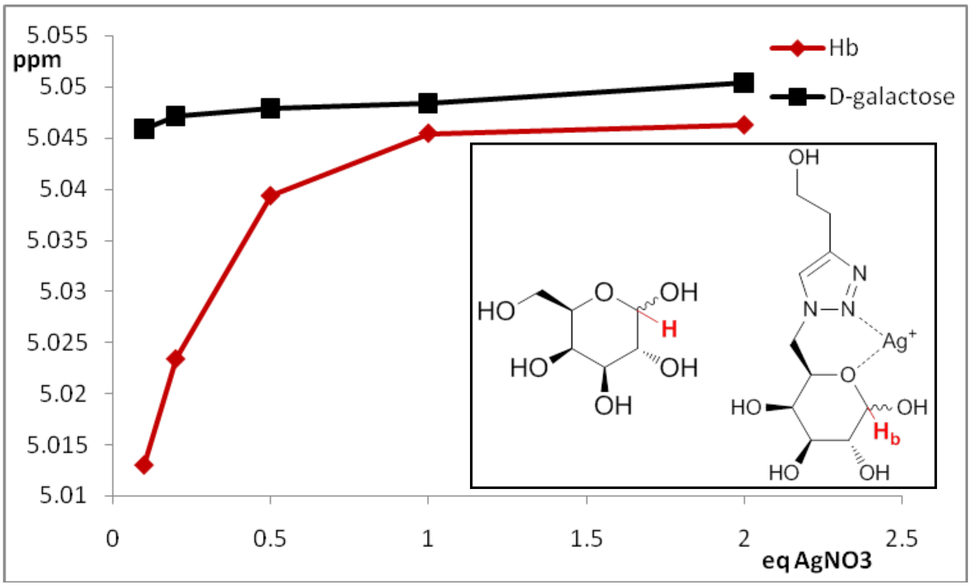

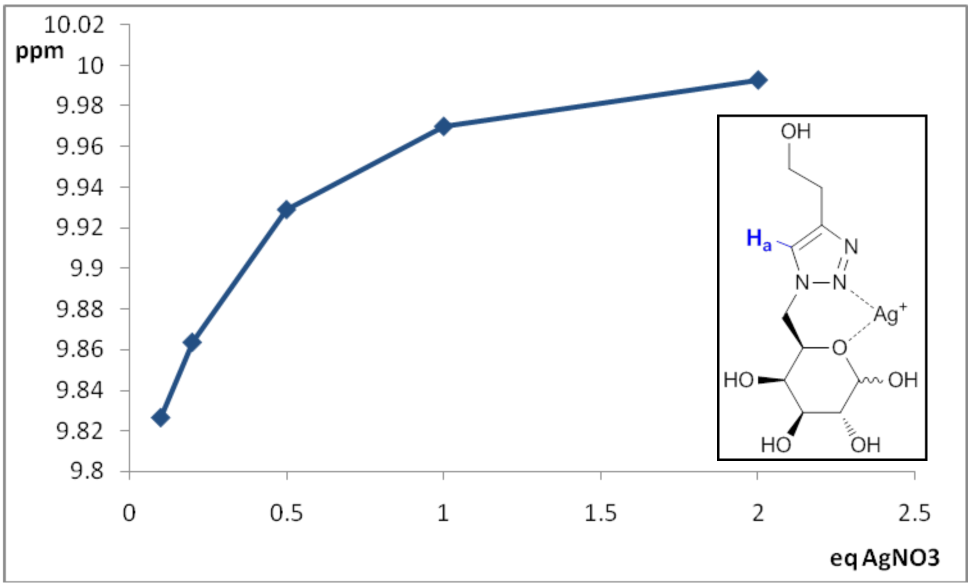

(b)

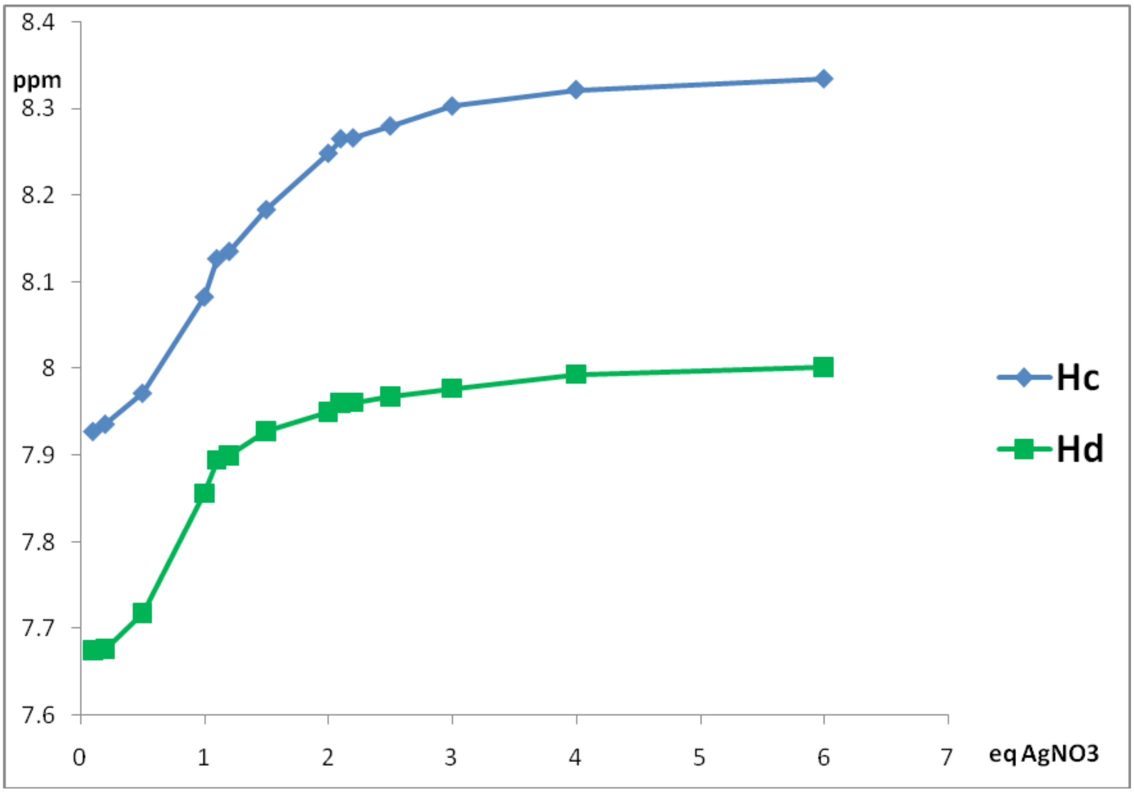
8.4
ppm
8.3
8.2
8.1
8
7.9
7.8
7.7
7.6
0
1
2
3
4
5
6
7
eq AgNO3
Hc
Hd

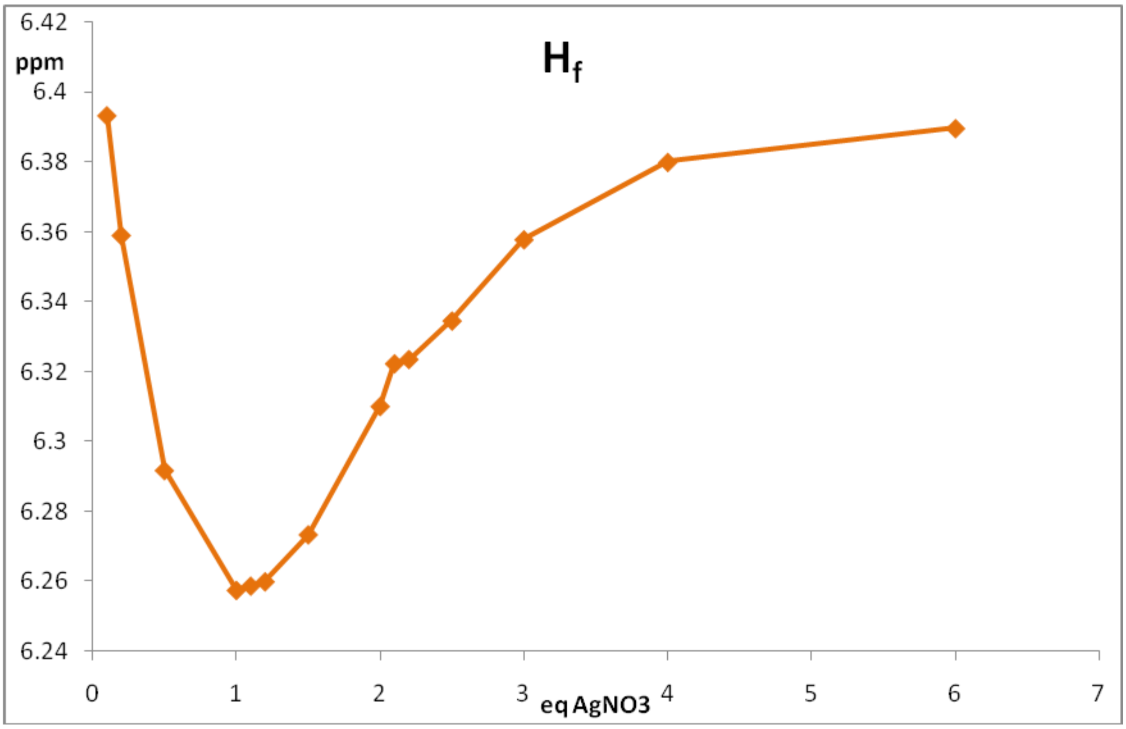
6.42
ppm
6.4
6.38
6.36
6.34
6.32
6.3
6.28
6.26
6.24
H_f
0
1
2
3
eq AgNO3
4
5
6
7

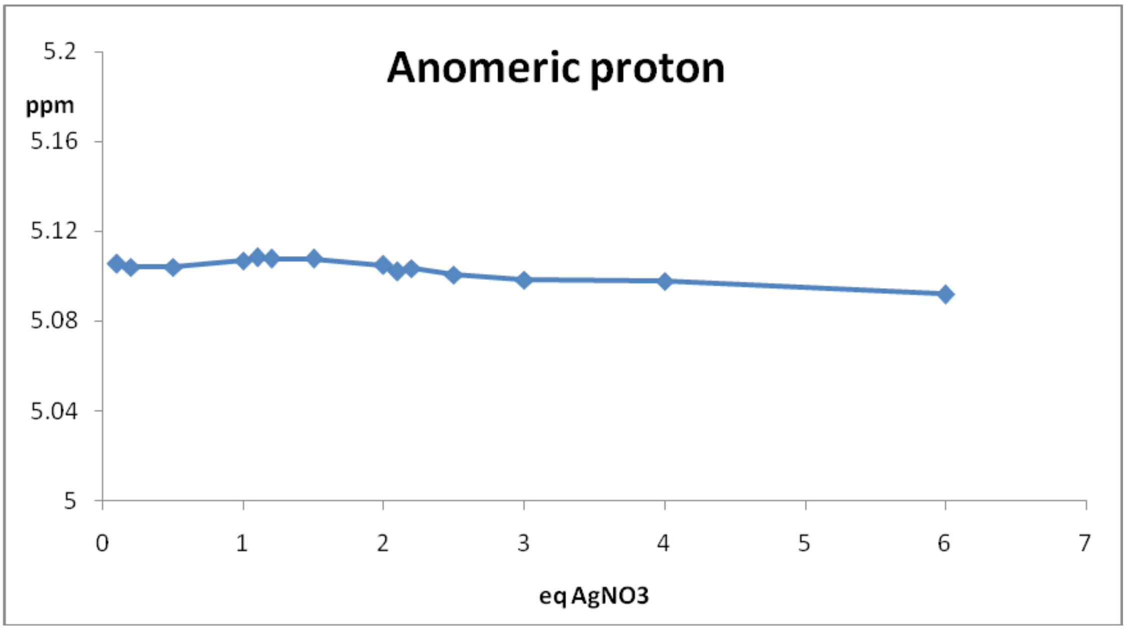

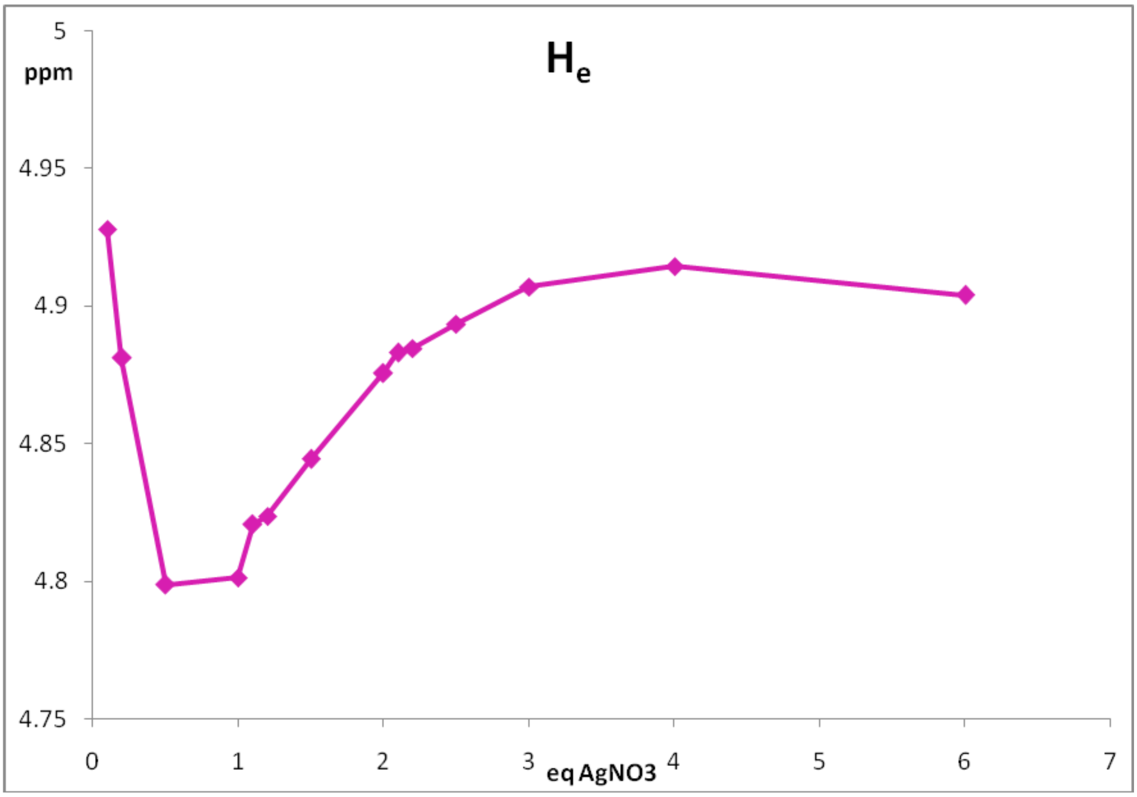

Figure S10: Job plot of the ^{1}H-NMR titration of **(a)** sugar (**1**) and D-galactose (4.2 mM) and **(b)** sugar (**2**) (2.0 mM) with $AgNO_3$ in D_2O (no data fitting was used).

NMR studies of AgNP formation

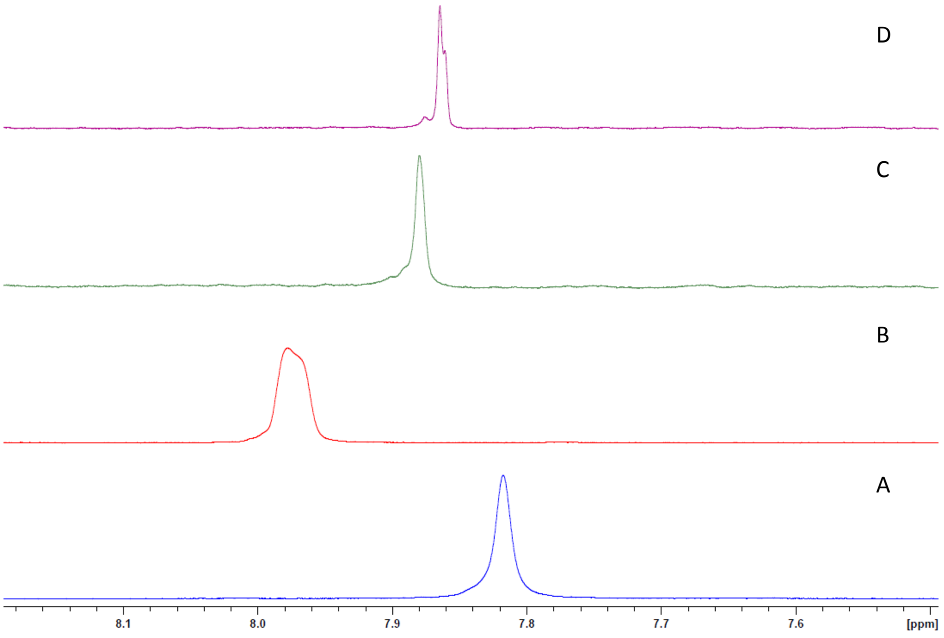

Figure S11.**A** Selected area compound (**1**) ^{1}H-NMR.**B**. Selected area of ^{1}H-NMR compound (**1**) + 1eq $AgNO_3$. **C**. Selected area of ^{1}H-NMR compound (**1**) + 1eq of Tollens reagent after 10 minutes. **D**. Selected area of ^{1}H-NMR compound (**1**) + 1eq of Tollens reagent after 24 hours.

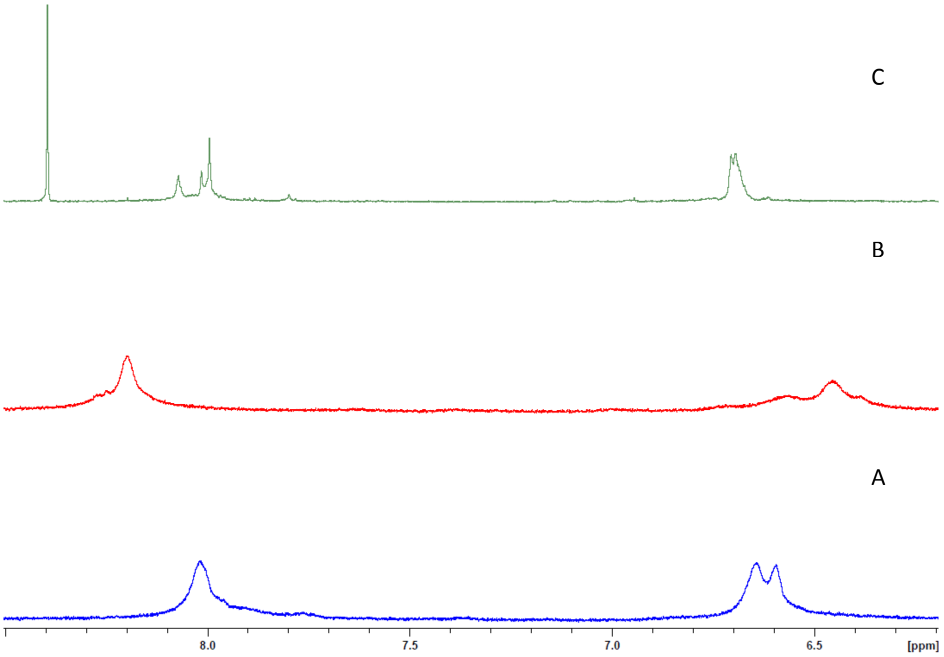

FigureS12 A. Selected area compound (**2**) ^{1}H-NMR.**B**. Selected area of ^{1}H-NMR compound (**2**) + 2eq $AgNO_3$. **C**. Selected area of ^{1}H-NMR compound (**2**) + 2 eq of Tollens reagent

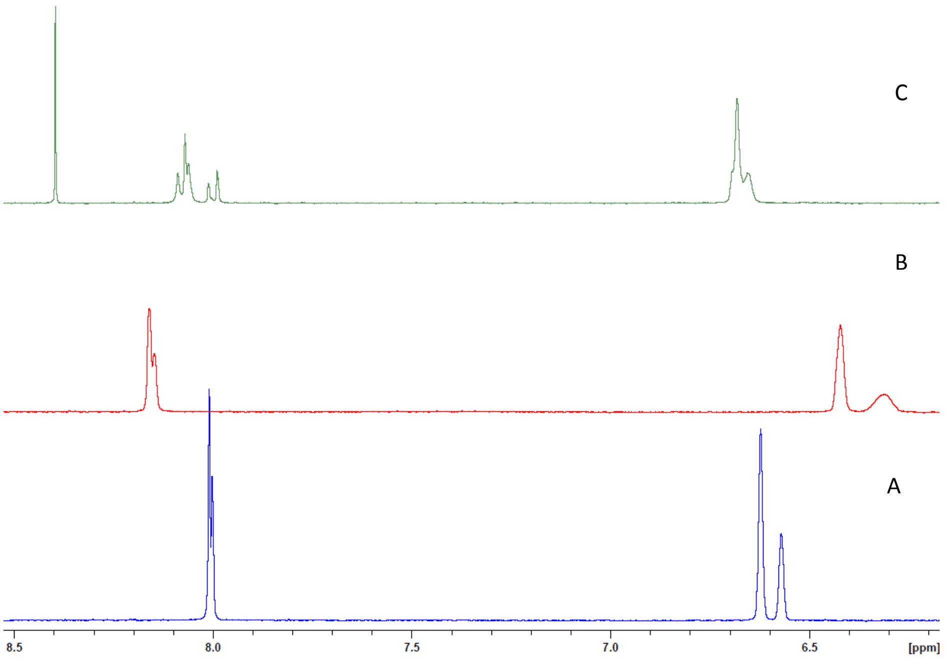

Figure S13 A. Selected area compound (**3**) ^{1}H NMR. **B**. Selected area of ^{1}H NMR compound (**3**) + 4eq $AgNO_3$. **C**. Selected area of ^{1}H NMR compound (**3**) + 4 eq of Tollens reagent

5.1 NMR studies of using (1)

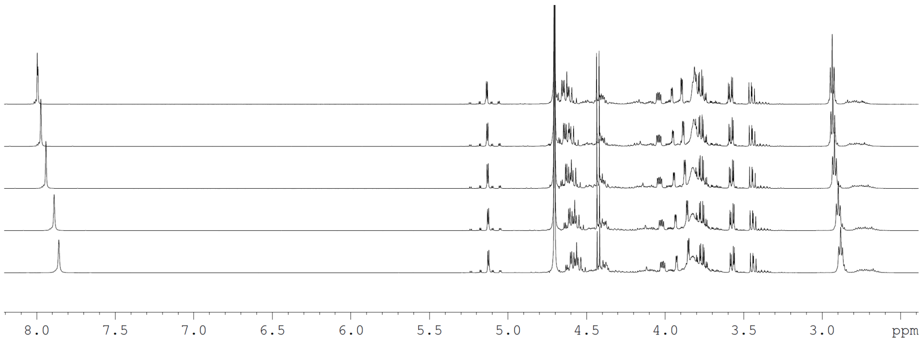

Figure S14. Stacking of 5 ^{1}H-NMR (500 MHz, D_2O) of (**1**) with 0.1, 0.2, 0.5, 1.0, 2.0 eq $AgNO_3$ (from bottom to top)

5.2 NMR studies of using (2)

(a)

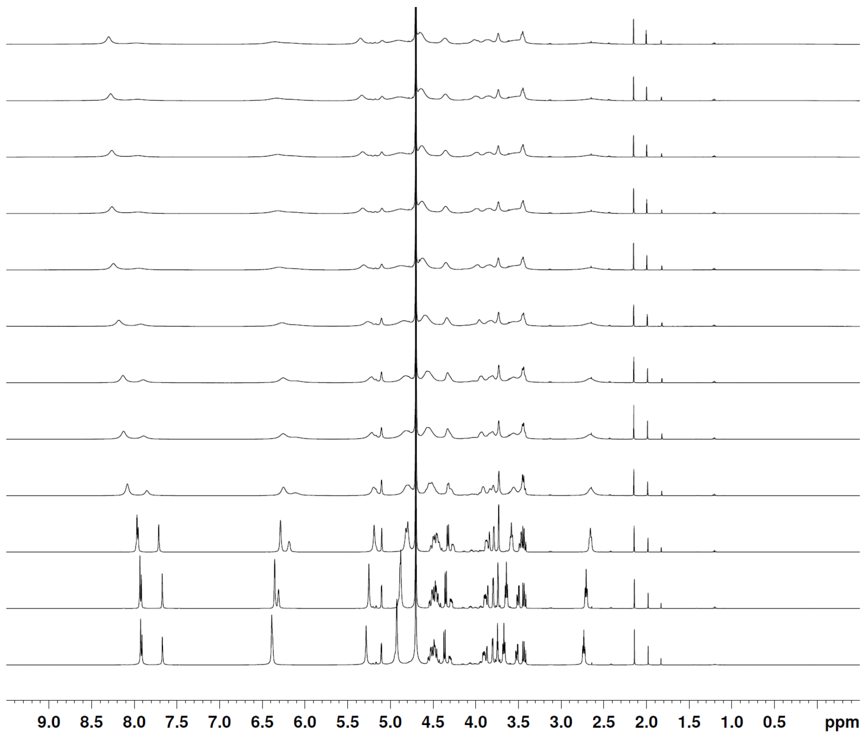

(b)

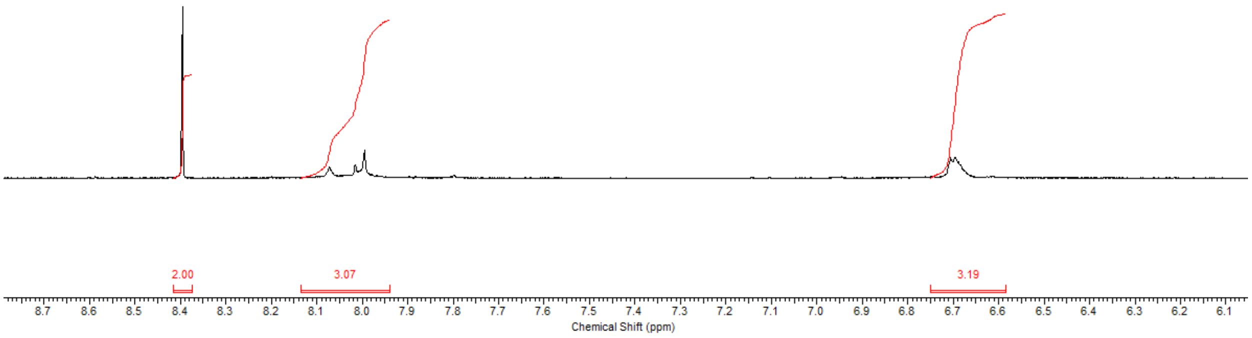

Figure S15. **(a)** Stacking of 14 ^{1}H-NMR (500 MHz, D_2O) of (**2**) with 0.1, 0.2, 0.5, 1.0, 1.1, 1.2, 1.5, 2.0, 2.1, 2.2, 2.5, 3, 4, 6 eq $AgNO_3$ (from bottom to top). **(b)** Selected area ^{1}H-NMR of (**2**) 68mM with 2eq Tollens showing integration of the aldehyde peak (2:3:3 ratio in relationship with the triazoles C-H and the resorcinol core C-H)

5.3 NMR studies of using (3)

(a)

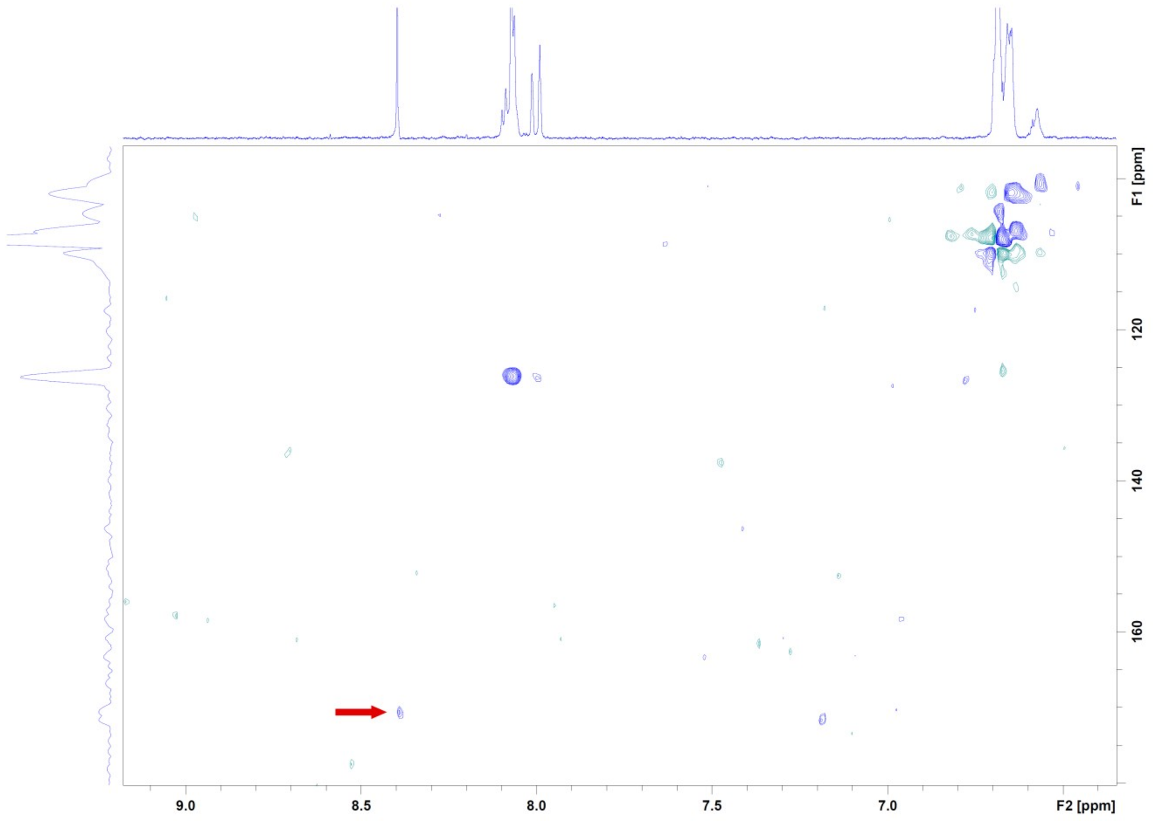

(b)

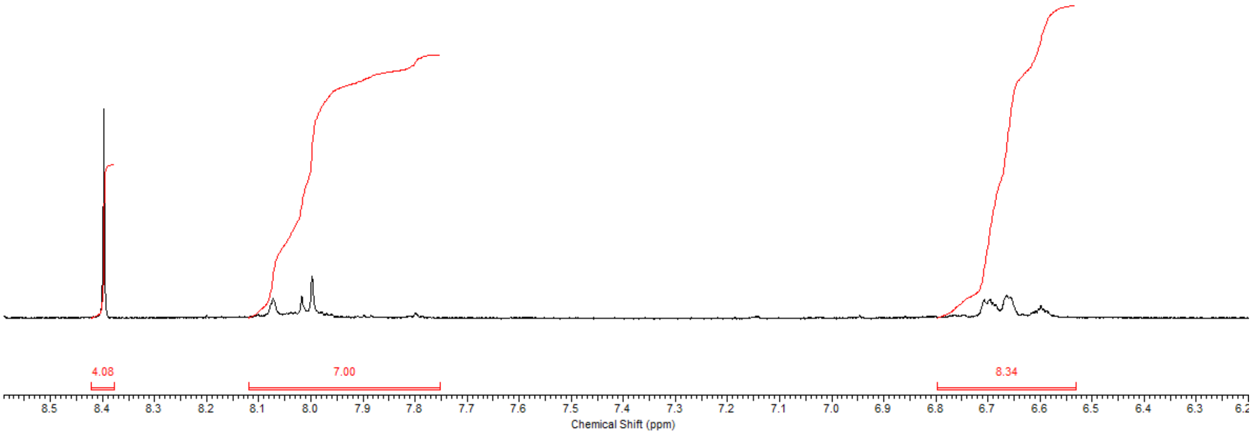

Figure S16. **(a)** HSQC of (**3**) 68mM with 2 eqTollens. Red arrow highlights peak of aldehyde at 8.39 ppm – 170.7 ppm. **(b)** Selected area ^{1}H-NMR of (**3**) with 4eq Tollens showing integration of the aldehyde peak (4:7:9 ratio in relationship with the triazoles C-H and the resorcinol cores C-H).

5.4 NMR studies of using D-galactose

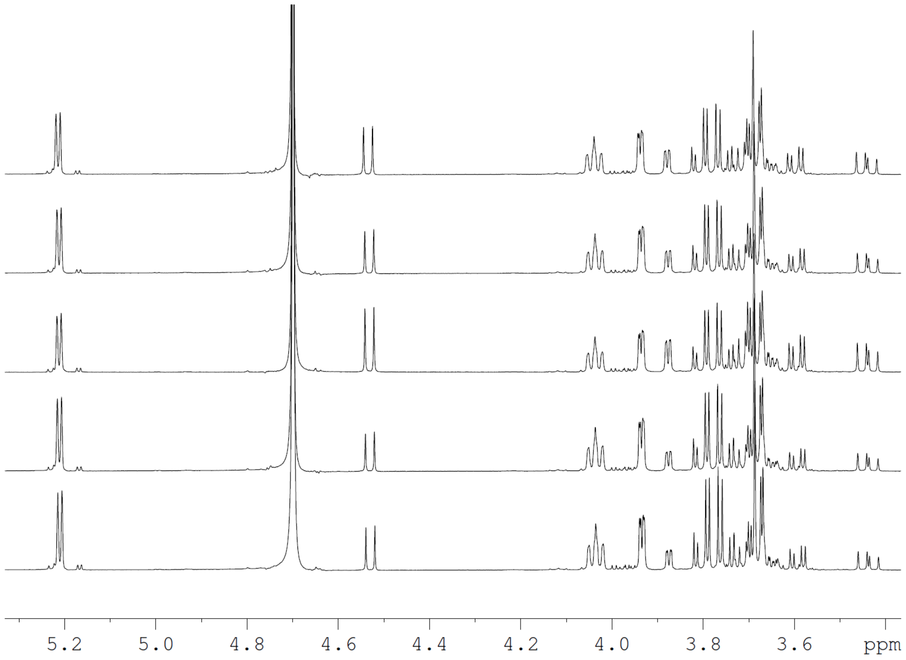

Figure S17. Stacking of 5 ^{1}H-NMR (400 MHz, D_2O) of D-galactose with 0.1, 0.2, 0.5, 1.0, 2.0 eq $AgNO_3$ (from bottom to top).

5.4 NMR studies using (4)

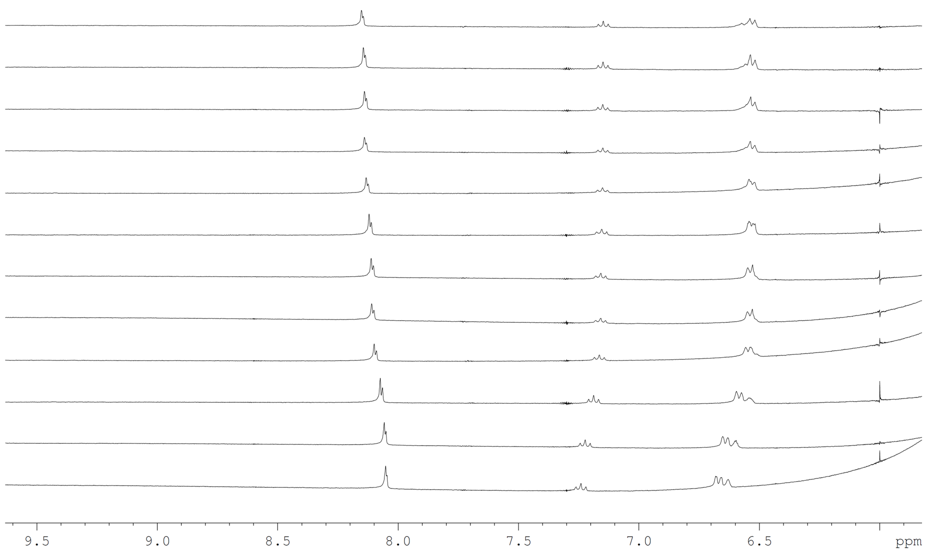

Figure S18. Stack plots of 12 selected areas ^{1}H-NMR (400 MHz, D_2O) of (**4**) with 0.1, 0.2, 0.5, 1.0, 1.1, 1.2, 1.5, 2.0, 2.1, 2.2, 2.5, 3 eq $AgNO_3$ (from bottom to top).

6.0 ES-MS analysis of (1)

All HR-MS measurements were made by direct injection into a Xevo ES$^+$-TOF high resolution mass detector using water as matrix of the sample and 10% MeOH in water as solution carrier continuously injected by a custom fitted micro-pump.

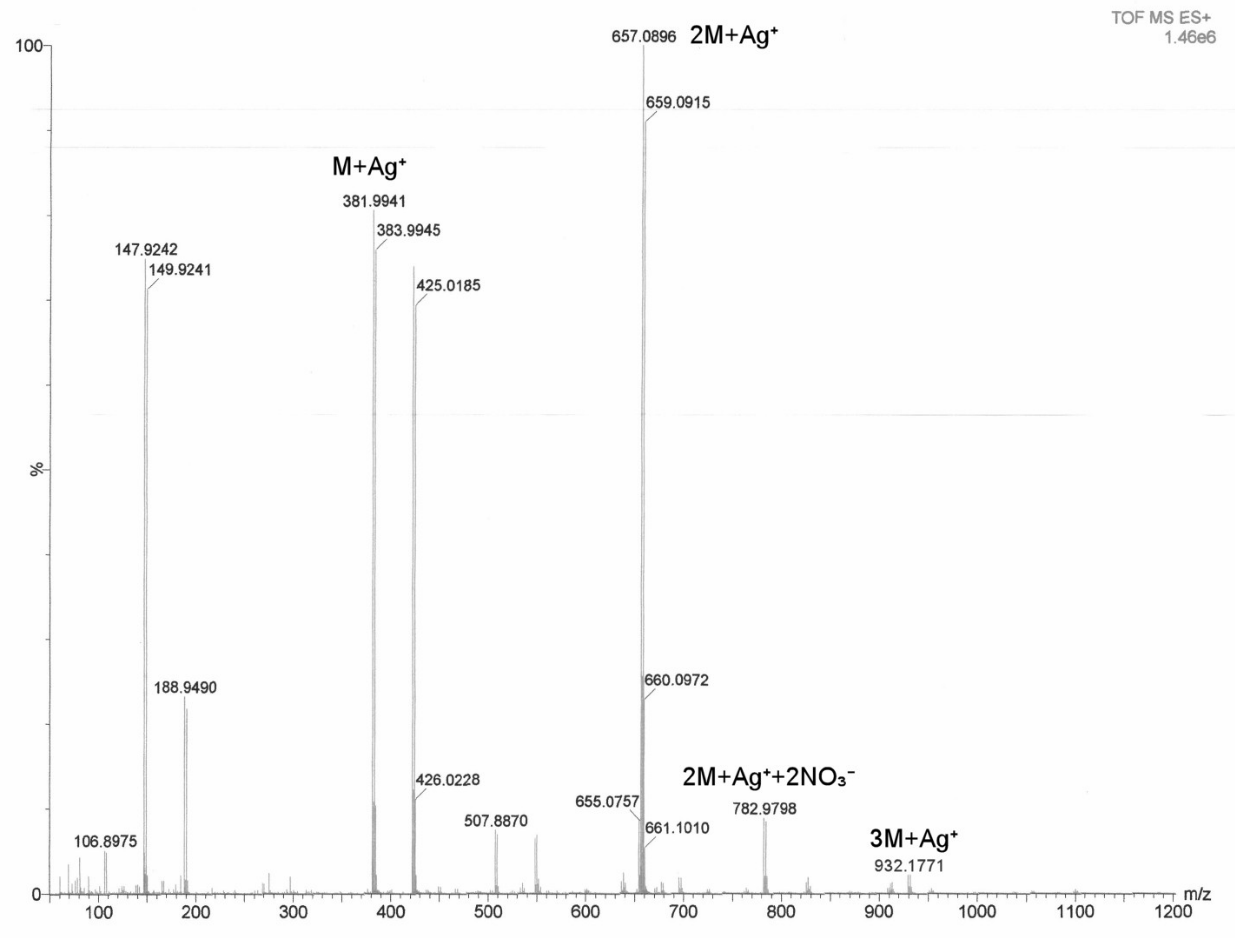

Figure S19. HRMS (ESI) of a solution of (**1**) with 1eq of $AgNO_3$ in H_2O.

6.1 ES-MS analysis of (2)

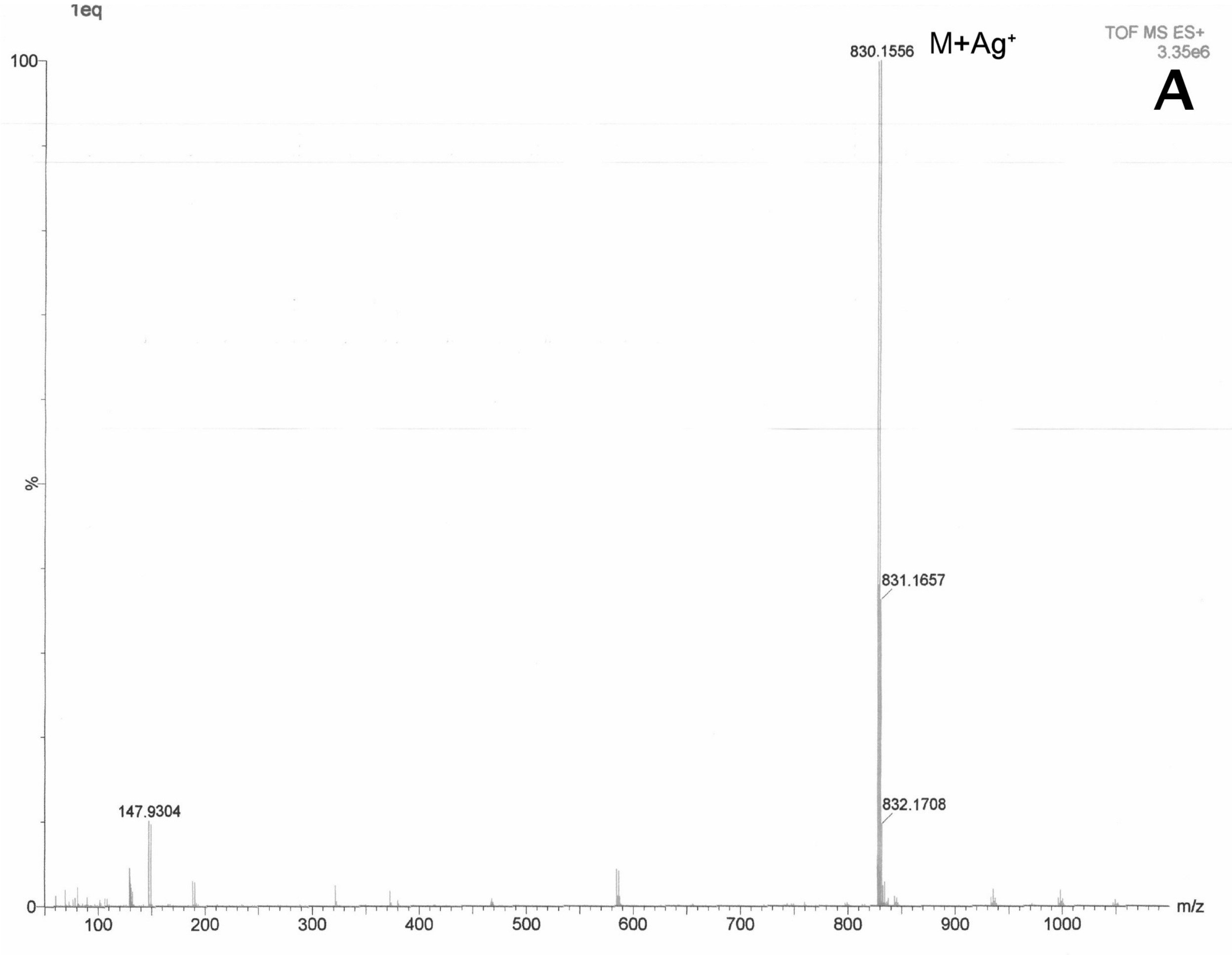

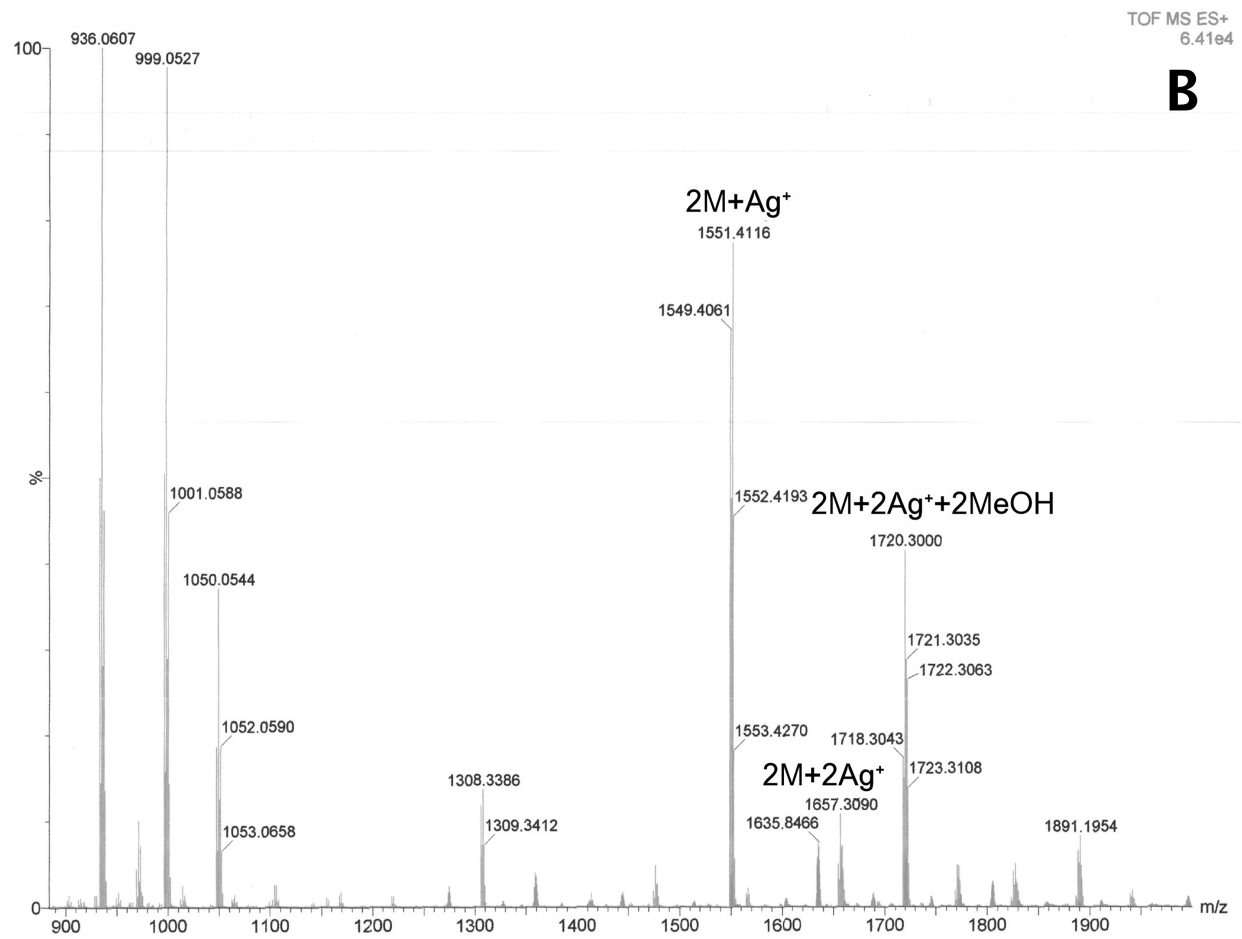

TOF MS ES+
6.41e4
B
100
%
0
936.0607
999.0527
1001.0588
1050.0544
1052.0590
1053.0658
1308.3386
1309.3412
2M+Ag⁺
1551.4116
1549.4061
1552.4193
1553.4270
2M+2Ag⁺+2MeOH
1720.3000
1721.3035
1722.3063
1718.3043
1723.3108
2M+2Ag⁺
1657.3090
1635.8466
1891.1954
m/z
900
1000
1100
1200
1300
1400
1500
1600
1700
1800
1900

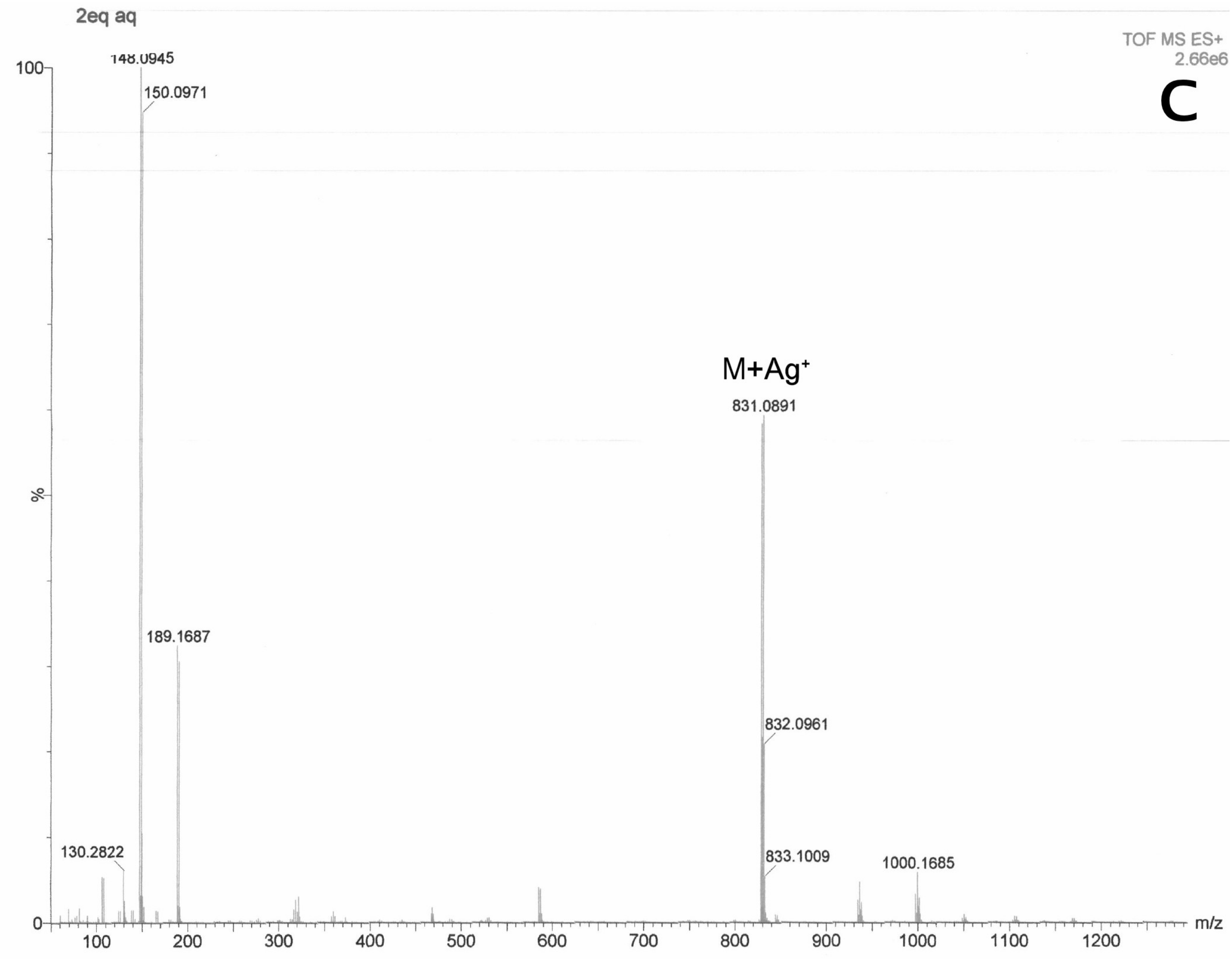
2eq aq
TOF MS ES+
2.66e6
C
148.0945
150.0971
189.1687
130.2822
M+Ag+
831.0891
832.0961
833.1009
1000.1685
100
%
0
m/z
100
200
300
400
500
600
700
800
900
1000
1100
1200

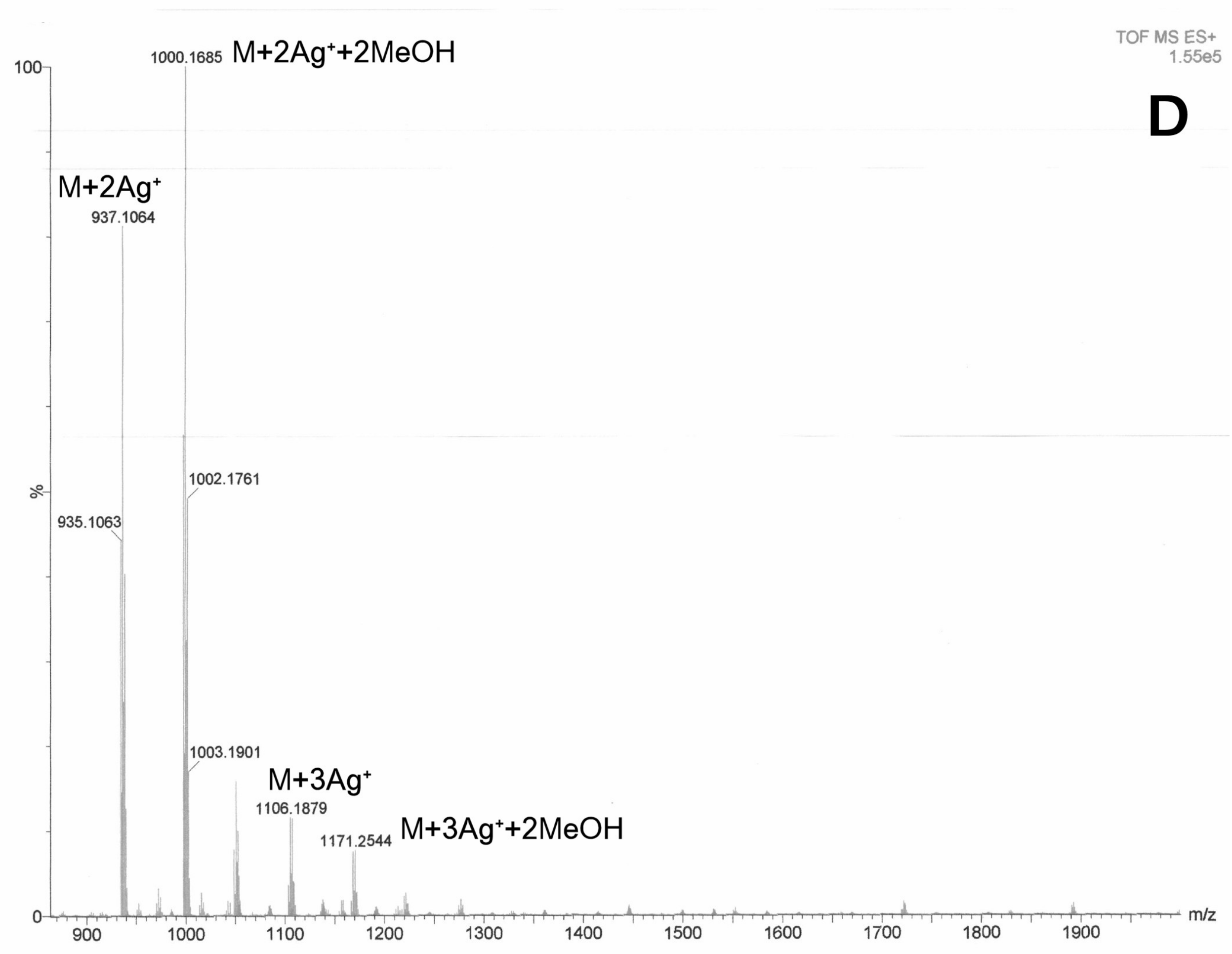

TOF MS ES+
1.55e5
D
100
%
0
1000.1685 M+2Ag⁺+2MeOH
M+2Ag⁺
937.1064
1002.1761
935.1063
1003.1901
M+3Ag⁺
1106.1879
1171.2544 M+3Ag⁺+2MeOH
900
1000
1100
1200
1300
1400
1500
1600
1700
1800
1900
m/z

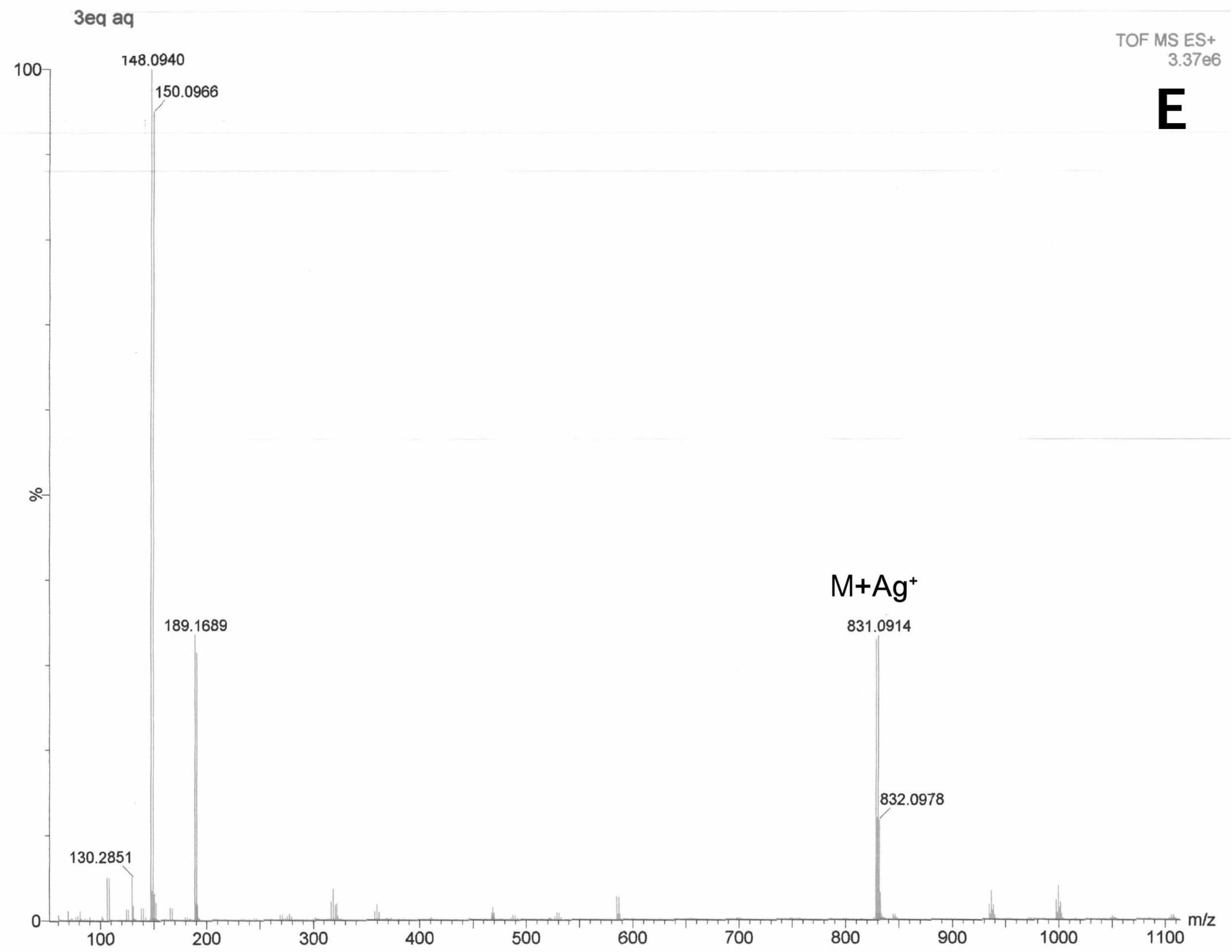
3eq aq
TOF MS ES+
3.37e6
E
148.0940
150.0966
189.1689
130.2851
M+Ag+
831.0914
832.0978
100
%
0
100
200
300
400
500
600
700
800
900
1000
1100
m/z

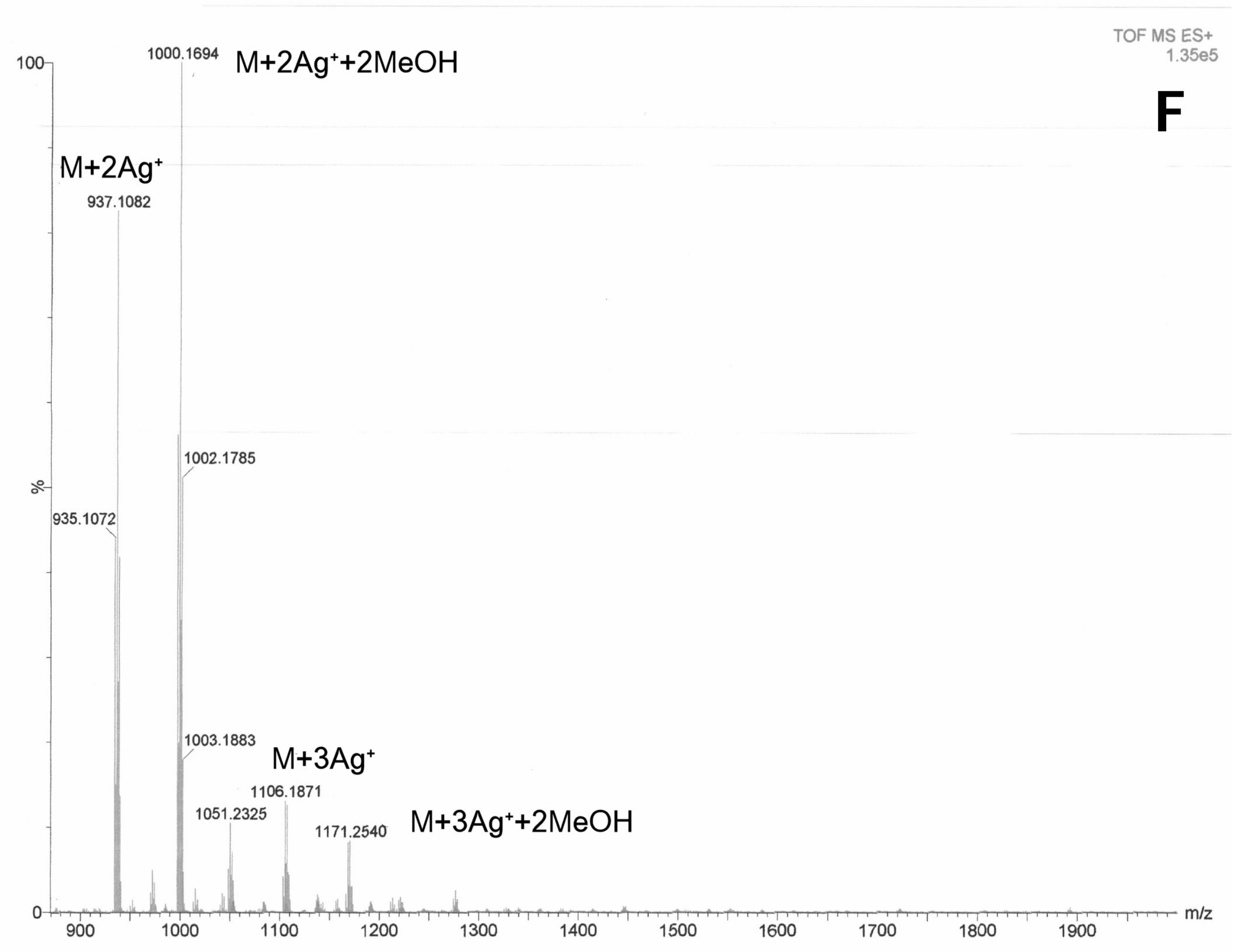

Figure S20. A-C-E: HRMS (ESI) of a solution of (**2**) with 1-2-3 eq of $AgNO_3$ in H_2O. **B-D-F:** Selected areas of HRMS (ESI) of a solution of (**2**) with 1-2-3 eq of $AgNO_3$ in H_2O expanded x20 to show details of higher order cluster formation.

7.0 References

[1] Burley, G. a; Gierlich, J.; Mofid, M. R.; Nir, H.; Tal, S.; Eichen, Y.; Carell, T. *Journal of the American Chemical Society*. **2006**, 128, 1398-9.

[2] Li, P.; Wang, L.; Zhang, Y. *Tetrahedron*. **2008**, 64, 10825-10830.
